# A replicable strategy for mapping air pollution’s community-level health impacts and catalyzing prevention

**DOI:** 10.1186/s12940-022-00879-3

**Published:** 2022-07-18

**Authors:** Philip J. Landrigan, Samantha Fisher, Maureen E. Kenny, Brittney Gedeon, Luke Bryan, Jenna Mu, David Bellinger

**Affiliations:** 1 Global Observatory on Pollution and Health, Boston College, Boston, MA USA; 2 Centre Scientifique de Monaco, Monaco, MC Monaco; 3 Environmental; Epidemiology Program, City University of New York, New York, USA; 4 Lynch School of Education and Human Development, Boston College, Boston, MA USA; 5 Morrissey College of Arts and Sciences, Boston College, Boston, MA USA; 6 Department of Neurology, Boston Children’s Hospital and Harvard Medical School, Boston, USA

**Keywords:** Air pollution, Global burden of disease, IQ, Community-level mapping, Neurodevelopmental disorders

## Abstract

**Background:**

Air pollution was responsible for an estimated 6.7 million deaths globally in 2019 and 197,000 deaths in the United States. Fossil fuel combustion is the major source.

**Hypothesis:**

Mapping air pollution’s health impacts at the community level using publicly available data and open-source software will provide a replicable strategy for catalyzing pollution prevention.

**Methods:**

Using EPA’s Environmental Benefits Mapping and Analysis (BenMAP-CE) software and state data, we quantified the effects of airborne fine particulate matter (PM_2.5_) pollution on disease, death and children’s cognitive function (IQ Loss) in each city and town in Massachusetts. To develop a first-order estimate of PM_2.5_ pollution’s impact on child IQ, we derived a concentration-response coefficient through literature review.

**Findings:**

The annual mean PM_2.5_ concentration in Massachusetts in 2019 was 6.3 μg/M^3^, a level below EPA’s standard of 12 μg/M^3^ and above WHO’s guideline of 5 μg/M^3^. In adults, PM_2.5_ pollution was responsible for an estimated 2780 (Confidence Interval [CI] 2726 – 2853) deaths: 1677 (CI, 1346 – 1926**)** from cardiovascular disease, 2185 (CI, 941–3409) from lung cancer, 200 (CI, 66–316) from stroke, and 343 (CI, 222–458) from chronic respiratory disease. In children, PM_2.5_ pollution was responsible for 308 (CI, 105–471) low-weight births, 15,386 (CJ, 5433-23,483) asthma cases, and a provisionally estimated loss of nearly 2 million Performance IQ points; IQ loss impairs children’s school performance, reduces graduation rates and decreases lifetime earnings. Air-pollution-related disease, death and IQ loss were most severe in low-income, minority communities, but occurred in every city and town in Massachusetts regardless of location, demographics or median family income.

**Conclusion:**

Disease, death and IQ loss occur at air pollution exposure levels below current EPA standards. Prevention of disease and premature death and preservation of children’s cognitive function will require that EPA air quality standards be tightened. Enduring prevention will require government-incentivized transition to renewable energy coupled with phase-outs of subsidies and tax breaks for fossil fuels. Highly localized information on air pollution’s impacts on health and on children’s cognitive function has potential to catalyze pollution prevention.

**Supplementary Information:**

The online version contains supplementary material available at 10.1186/s12940-022-00879-3.

## Introduction

Air pollution – unwanted material released to the atmosphere by human activity - is the world’s largest environmental cause of disease, disability and premature death [1]. Air pollution was responsible for an estimated 6.7 million (CI, 5.9–7.5 million) deaths globally in 2019, with the overwhelming majority in low-income and middle-income countries. In the United States, air pollution is responsible for an estimated 197,000 deaths annually (95% CI, 183,000 – 214,000) [2]. Fossil fuel combustion is the predominant anthropogenic source of ambient air pollution. It is responsible for 85% of all airborne particulate pollution and virtually all pollution by oxides of nitrogen (NO_X_) and sulfur (SO_X_) [3].

Airborne fine particulate matter (PM_2.5_) air pollution is linked to multiple non-communicable diseases [4]. In adults, these include cardiovascular disease, stroke, chronic obstructive pulmonary disorder, lung cancer and diabetes [4–11]. In infants and children, air pollution increases risk for premature birth [12–14], low birthweight [12–14], stillbirth [12–14], asthma [15–19], and impaired lung development [18–20]. Prematurity and low birth weight are risk factors for cardiovascular disease, kidney disease, hypertension and diabetes in adult life [21]. Impaired lung growth increases risk for chronic respiratory disease [20].

Emerging evidence indicates that air pollution is associated with neurologic dysfunction in both adults and children [22–39]. In adults, associations are reported between PM_2.5_ pollution and risk of dementia [22–27]. In children, exposures to PM_2.5_ and other components of air pollution are linked to loss of cognitive function (IQ loss), memory deficits, behavioral dysfunction, reductions in brain volume and increased risks of attention deficit/hyperactivity disorder (ADHD) and autism spectrum disorder (ASD) [27–39].

All of these adverse health effects occur at PM_2.5_ exposure levels below the US Environmental Protection Agency’s current annual mean standard of 12.0 μg/m^3^ [5, 40, 41]. Recognizing that PM_2.5_ pollution causes adverse health effects at levels previously thought to be safe, the World Health Organization recently lowered their recommended PM_2.5_ guideline from 10 μg/m^3^ to 5 μg/m^3^ [4].

Air pollution and its health effects are not equitably distributed. Multiple studies document that poor, minority and marginalized communities bear a disproportionately heavy burden of air pollution exposure and pollution-related disease [42–48]. In the COVID-19 pandemic, minority communities exposed to high levels of particulate pollution experienced disproportionately increased rates of hospitalization and death [49].

Air pollutant emissions have decreased by 74% In the United States since passage of the Clean Air Act in 1970 [50]. Air pollution control has proven highly cost-effective, yielding an estimated return of $30 for every dollar invested [51]. The consequences have been improved health, reduced pollution-related disease and death and increased longevity [6]. A particularly noteworthy triumph was control of airborne lead pollution by the removal of lead from gasoline. This intervention resulted in a more than 95% reduction in mean blood lead levels in American children and in an estimated 5-point gain in the average IQ of every child born in the United States since 1980 [52].

A challenge to the continuing control of air pollution in the aftermath of these gains is that pollutant levels in high-income countries are much lower today than in the past and pollution’s health impacts may not be immediately visible. In this circumstance there is danger that pollution will be regarded as a solved problem and that progress against pollution will stall.

A strategy for overcoming such complacency is to use newly developed open-source software and publicly available data to quantify air pollution’s health impacts at a local community level [53–55]. Findings from such localized mapping can be brought to the attention of the public and policy makers. This information has potential to increase awareness of the immediacy of pollution’s continuing health threats and thus to mobilize citizen action and catalyze pollution prevention.

In this report, we describe a study that uses publicly available data and open-source software **to** estimate the impacts of ambient PM_2.5_ air pollution on disease, death and children’s cognitive impairment (IQ loss) at the local level in each city and town across Massachusetts. This highly granular approach can be replicated in other areas of the United States.

## Methods

### Air pollution levels

To estimate fine airborne particulate matter (PM_2.5_) pollution exposures in Massachusetts, we used 2019 data from the Massachusetts Department of Environmental Protection’s (DEP) Ambient Air Quality Monitoring Network - a web of 22 air quality-monitoring stations dispersed across the state. When air pollution data were available within a city or town, we used these data to estimate air pollution exposures in the town. When town-specific data were not available, we applied data for the surrounding county. In the case of towns for which no county-level data available were available, we applied data from the nearest adjacent county. Thus, for four of the 14 counties - Norfolk, Barnstable, Dukes, and Nantucket – no monitoring data were available. Therefore, for these counties, we used the values in contiguous counties to estimate pollution levels. For instance, Norfolk County shares borders with Bristol, Worcester, Middlesex, Suffolk, and Plymouth counties. The mean PM2.5 concentration in these five counties, 6.79 μg/m^3^, was used to estimate exposure in Norfolk County. Barnstable County shares a border with Plymouth County, and the PM2.5 concentration for Plymouth County was applied to Barnstable County. Dukes and Nantucket Counties are both islands, and the value for the closest mainland county, Plymouth County, was therefore assumed for these counties.

### Demographic and health data

We obtained demographic data on the size and structure of the population of each city and town in Massachusetts in 2019 from the US Census Bureau. We obtained data on incidence and mortality of heart disease, stroke, chronic obstructive pulmonary disease (COPD), and diabetes as well as data on pediatric asthma in each city and town from the Massachusetts Department of Public Health’s Population Health Information Tool [56]. We obtained data on incidence and mortality of lung and bronchial cancer from the Massachusetts Cancer registry [57]. We obtained data on the numbers of low birthweight babies and premature births in each city and town in Massachusetts from the Annie E. Casey Foundation’s Kids Count Data Center [58].

### The burden of disease attributable to air pollution

We estimated the burden of disease and death attributable to air pollution in each city and town in Massachusetts using the US Environmental Protection Agency’s open-source Environmental Benefits Mapping and Analysis Program (BenMap-CE) [59]. BenMap-CE is software that contains data on air pollution levels, demographic data, and a range of concentration-response coefficients derived from epidemiologic studies quantifying relationships between air pollution exposure levels and adverse health effects (Table 1). These concentration-response coefficients specify the health impacts associated with each 1μg/m^3^ increase in PM_2.5_ concentration [59, 63]. In our analyses, we set a counterfactual PM_2.5_ exposure level 0 μg/m^3^. BenMap-CE supports the development of estimates of the burden of disease attributable to air pollution and the creation of maps showing air pollution concentrations and pollution-related health effects at the community level.

**Table 1:** Concentration-response coefficients for disease outcomes associated with PM_2.5_ exposure

Health indicator	Study author	Concentration-response coefficient (confidence interval)
All-cause mortality	**Di et al. (2017)** [41]	0.00704 (0.0069–0.00723)
**Adult diseases**		
Stroke	**Lin et al. (2017)** [60]	0.0122 (0.0039–0.0198)
COPD/CLRD	**Lin et al. (2018)** [61]	0.01906 (0.012–0.026)
Heart Disease	**Krewski et al. (2009)** [62]	0.0215 (0.017–0.025)
Lung Cancer	**Krewski et al. (2009)** [62]	0.013 (0.0055–0.021)
**Pediatric diseases**		
Childhood asthma incidence	**Khreis et al. (2017)** [15]	0.030 (0.0099–0.0487)
Low Birth Weight	**Sun et al. (2016)** [14]	0.009 (0.003–0.014)

To examine the relationship between PM_2.5_ pollution level and death from all causes (all-cause mortality), we used the concentration-response function from Di et al. [41] To examine relationships between PM_2.5_ pollution and specific disease outcomes, we used the concentration-response functions indicated in Table 1.

### Air pollution and IQ loss in children

Information on the relationship between PM_2.5_ air pollution and brain development in children is still emerging [27–39]. A concentration-response coefficient based on a meta-analysis correlating PM_2.5_ concentrations to IQ loss has not yet been described in the literature nor is such a coefficient is included in EPA’s BenMap-CE software. Therefore, to derive a provisional concentration-response coefficient that could provide a first-order estimate of the relationship between airborne PM_2.5_ air pollution levels and cognitive loss in Massachusetts children, we conducted a review of the world’s literature to identify reports that had examined relationships between air pollution and loss of neurocognitive function, including IQ loss in infants and children.

We sought articles in all languages examining relationships between air pollution and IQ loss. The databases included were PubMed, Scopus and Embase. Our search string included the terms: “air pollution”, “fine particulate matter”, “PM2.5”, “ozone”, “nitrogen dioxide”, “black carbon”, “polycyclic aromatic hydrocarbon”, “PAH”, “second-hand smoke”, “household air pollution”, “cognitive function”, “intelligence”.” IQ” “autism”, “ADHD”, “neurodevelopment”, “neurotoxicity”, “infant”, “child”, “adolescent” and “prenatal”.

We screened search results by title and then by abstract to identify relevant articles that met our inclusion criteria. We excluded nonhuman studies, non-original studies, reviews, and studies that did not quantify associations between PM_2.5_ air pollution and cognitive endpoints. We excluded articles examining environmental exposures other than air pollution. We used data extracted from this review to develop a provisional concentration-response coefficient quantifying the IQ loss in children that is associated with each unit increase in PM_2.5_ pollution.

Our literature review identified 1169 published articles that could potentially support development of an exposure-response coefficient. After removing duplicates, we identified 770 unique studies that met our screening criteria. We eliminated 671 of these studies through reviewing abstracts and determining that they did not meet our inclusion criteria. We eliminated another 72 studies that were not relevant to our investigation. At the conclusion of our review, we were left with 27 original studies that had quantitatively examined relationships between air pollution and neurocognitive impairment in children.

Three of these studies (Harris et al., 2015 [37]; Porta et al., 2016 [38]; Wang et al., 2017 [39]) most closely met our inclusion/exclusion criteria. They each found negative associations between PM_2.5_ pollution concentrations in prenatal and/or early postnatal life and IQ loss in children, and the association achieved statistical significance in the report by Wang et al. While both Verbal and Performance IQ were negatively affected by air pollution, strongest associations were consistently seen in all three studies between PM_2.5_ pollution levels and loss of Performance IQ (PIQ). Additionally, the study by Wang et al. reported effect modification by socio-economic status, with the inverse association between PM_2.5_ and Performance IQ stronger among less advantaged children [39].

We took the estimates of Performance IQ (PIQ) loss associated with each 1 μg/m^3^ increase in PM_2.5_ concentration in the three studies that met our inclusion criteria and weighted them by sample size. This calculation produced a provisional estimate of 0.41 PIQ points lost in children for each 1 μg/M^3^ increase in the ambient PM_2.5_ concentration (Table 2).

**Table 2:** Development of a provisional concentration-response coefficient relating PM_2.5_ air pollution concentration (μg/m^3^) with Performance IQ (PIQ) loss in children

Study	Sample size	Age at exposure	PIQ points lost loss for each 1 μg/m^3^ increase in PM_2.5_
**Harris et al. (2015)** [37]	1109	Prenatal	−0.16
**Porta et al. (2016)** [38]	474	Prenatal and childhood (1–7 years)	−0.40
**Wang et al. (2017)** [39]	1087	Childhood (9–11 years)	−0.61
**COMBINED**	2670	–	**−0.41**

To develop a provisional, first-order estimate of the PIQ loss caused by PM_2.5_ air pollution in children 0–9 years of age, we applied this concentration-response coefficient to air pollution and demographic data for each city and town in Massachusetts. We assumed that the concentration-response coefficient was linear in form and that it extended down to a PM_2.5_ pollution level of zero. This estimate can be reconsidered in the future when a published concentration-response coefficient based on a meta-analysis becomes available.

## Results

### Air pollution levels in Massachusetts

**Fig. 1:**
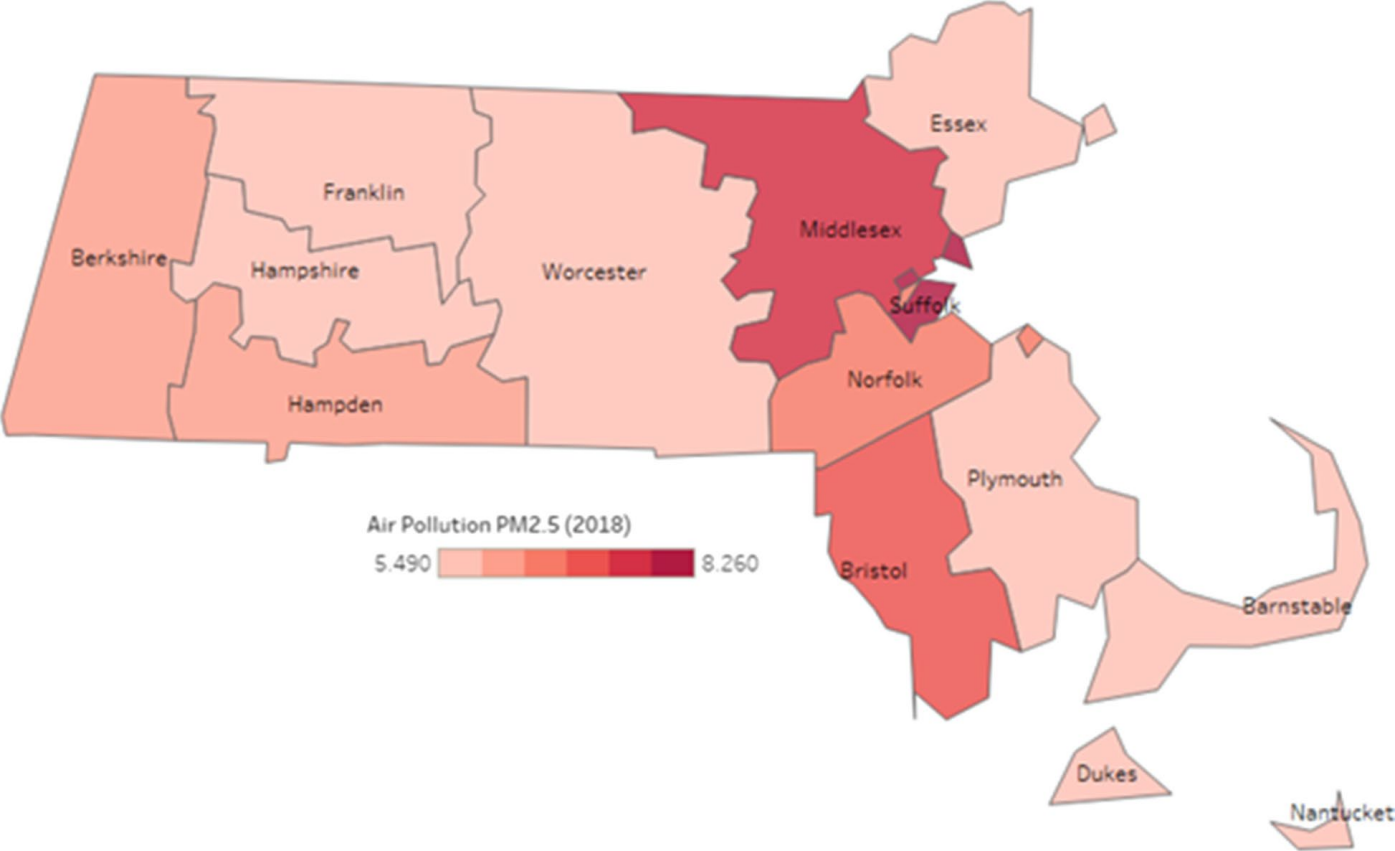
PM_2.5_ Concentration by County, Massachusetts, 2019. Source: Massachusetts Department of Environmental Protection (DEP) Ambient Air Quality Monitoring Network

The annual mean concentration of PM_2.5_ pollution in Massachusetts in 2019 was 6.3 μg/M^3^. This concentration is below the US Environmental Protection Agency’s annual mean PM_2.5_ standard of 12 μg/M^3^ [40], but above the World Health Organization’s recommended guideline of 5 μg/M^3^ [4]. Air pollution levels by county are shown in Fig. 1.

Massachusetts sources released 938,201 tons of air pollutants in 2017, the most recent year for which data are available [64, 65]. Approximately 70% of these emissions (655,191 tons, including 3645 tons of PM_2.5_) were produced by mobile sources – cars, trucks, buses, trains, ships and planes – while the remaining 30% (283,010 tons, including 21,539 tons of PM_2.5_) were discharged by stationary sources including electricity-generating plants, other industrial facilities and buildings [64].

Virtually all of the air pollution produced in Massachusetts results from the combustion of fossil fuels. Massachusetts’ continuing heavy dependence on fossil fuels for power generation, heating and transport is sustained by multi-billion-dollar subsidies and tax breaks provided by state and federal governments to the fossil fuel industry [66].

### Massachusetts demographics

The estimated population of Massachusetts on July 1, 2019 was 6,892,503 persons [67]. By gender, 51.5% of the population is female. By age, 5.2% of the population is under 5 years of age, 19.6% is under 18 years of age, and 17.0% is 65 years of age and above. By race, 80.6% of the Massachusetts population is White alone, 9.0% is Black or African-American alone, 0.5% is American Indian and Alaska Native alone, 7.2% is Asian alone, and 0.1% of is Native Hawaiian and Other Pacific Islander alone. 2.6% of the population identifies as belonging to two or more races. 12.4% of the population identifies as Hispanic or Latino.

### Deaths due to air pollution in Massachusetts

We estimate that air pollution was responsible for 2780 deaths in Massachusetts in 2019 (Confidence Interval [CI], 2726 – 2853), nearly 5% of the 58, 557 deaths in the state. Lung cancer was the largest cause of air-pollution-related death in Massachusetts in 2019, responsible for 2185 deaths (Confidence Interval [CI], 941–3409), followed by heart disease (1677 deaths, CI: 1346 – 1926), chronic lower respiratory disease (343 deaths, CI: 222–458) and stroke (200 deaths, CI: 66–316). We estimate additionally that the births of 308 babies of low birth weight (CI: 105–471) and 15,386 cases of pediatric asthma (CI, 5433 – 23,483) were attributable to air pollution (Table 3).

**Table 3:** Estimated adult deaths and pediatric disease cases attributable to PM_2.5_ air pollution by cause, Massachusetts, 2019

Cause	Deaths and disease cases (number and confidence interval)
**Adult Deaths - All Causes**	**2780 (2726 – 2853)**
**Adult Deaths by Cause of Death**	
Chronic lower respiratory disease	**343 (222–458)**
Heart DISease	**1677 (1346 – 1926)**
Lung, Tracheal, and Bronchial Cancer	**2185 (941–3409)**
Stroke	**200 (66–316)**
**Pediatric diseases**	
Low birth weight	**308 (105–471)**
Pediatric asthma	**15,386 (5433 – 23,483)**

*Note:* The sum of PM_2.5_-related deaths due to specific causes is greater than the number of PM_2.5_-related deaths from all causes combined, because different exposure-response coefficients from different studies were used in calculating deaths from each specific cause

### Deaths due to air pollution in the cities and towns of Massachusetts

**Fig. 2:**
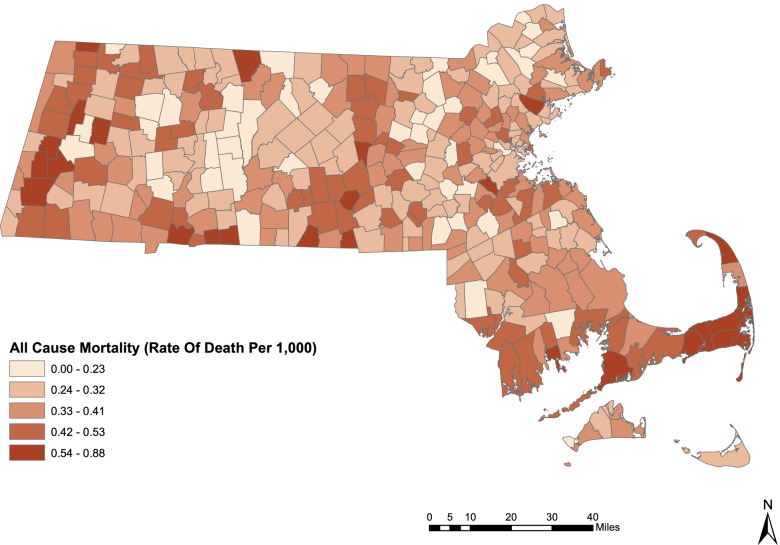
All-Cause Mortality (Deaths per 1000) Attributable to PM_2.5_ Air Pollution by City and Town, Massachusetts, 2019

**Fig. 3:**
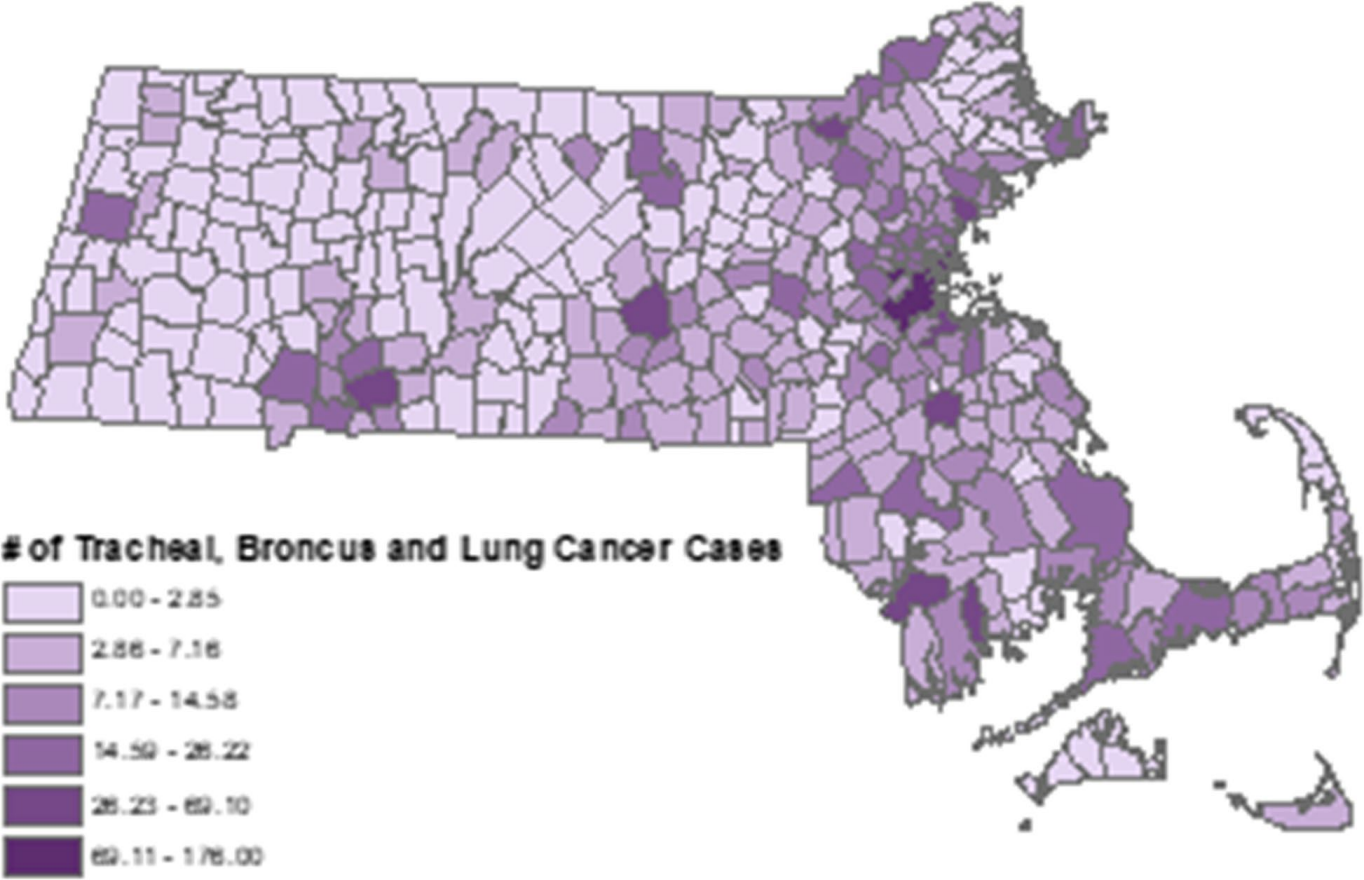
Lung Cancer Deaths Attributable to PM_2.5_ Air Pollution by City and Town, Massachusetts, 2019

PM_2.5_ air pollution was responsible for premature deaths in every county, in every city and in all but the very smallest towns in Massachusetts in 2019 (Figs. 2 and 3 and Supplementary Tables 5 and 6).

PM_2.5_ Air Pollution and IQ Loss in Massachusetts Children. We estimate that early-life exposures to PM_2.5_ air pollution were associated with the loss of nearly 2 million Performance IQ points lost among children 0–9 years of age in Massachusetts in 2019, an average loss of more than 2 points per child. Information on IQ loss by city and town is presented in Supplementary Table 7.

## Discussion

We found in this analysis that PM_2.5_ air pollution was responsible for an estimated 2780 (CI: 2726 – 2853) deaths in Massachusetts in 2019, nearly 5% of all deaths in the state. Of these deaths, an estimated 1677 were due to cardiovascular disease, 2185 to lung cancer, 200 to stroke, and 343 to chronic lower respiratory disease. Air pollution was responsible additionally for the birth of 308 low-birthweight babies (5.5 lbs. or less) and for 15,386 cases of pediatric asthma.

The annual mean concentration of PM_2.5_ air pollution in Massachusetts in 2019 was 6.3 μg/M^3^. Thus, the adverse health effects we estimated occurred at exposure levels below the US Environmental Protection Agency’s annual PM_2.5_ standard of 12 μg/M^3^ [40]. An extensive body of literature documents that PM_2.5_ pollution exposures at concentrations below 12 μg/M^3^ are associated with adverse health effects [4, 5, 41].

Open-source software enables quantification of air pollution’s health effects at a community level and supports mapping of localized health effects [53–55]. Using US EPA’s BenMAP-CE software [59], we documented that disease and death caused by air pollution occur in every community in Massachusetts. No community in the state is immune to air pollution’s impacts regardless of size, location, demographics, socioeconomic status or median family income. Air pollution does not respect political boundaries.

Air pollution’s health effects are not evenly distributed. Multiple studies have documented disproportionately high concentrations of pollution and disproportionately heavy burdens of pollution-related disease and death in economically disadvantaged and socially vulnerable communities [42–48]. This inequitable distribution of hazardous environmental exposures and polluti0on-related disease is an example of environmental injustice and a consequence of structural racism. It reflects long-standing inequities in the valuation of real estate (“red-lining”) and the deliberate siting of highways and polluting industrial facilities in low-income, predominantly minority communities [47, 48].

Prompted by new data documenting the negative effects of PM_2.5_ pollution exposure in early life on brain development [27–39], we estimated air pollution’s impact on cognitive function in Massachusetts children. To support this analysis, we derived a provisional concentration-response coefficient linking PM_2.5_ concentrations to IQ loss through review of the published literature. Applying this coefficient to PM_2.5_ air pollution and demographic data for each city and town in Massachusetts**,** we estimated provisionally that air pollution is responsible for the loss of nearly 2 million Performance IQ points in Massachusetts children 0–9 years of age, an average loss of more than 2 Performance IQ points per child.

Robust cognitive function is essential for individual success and societal survival in today’s post-industrial, knowledge-driven world [68]. Cognitive function is a key predictor of earning potential, health and longevity as well as a core underpinning of the human capital of cities and countries [68, 69]. The Intelligence Quotient (IQ score) is the most widely used index of cognitive function [70]. IQ has the advantage that it is a highly standardized measure that has been used extensively in studies documenting the effects of toxic environmental exposures such as lead, mercury and pesticides on children’s brain development and cognitive function [71–73].

The IQ score measures both verbal and non-verbal cognitive abilities. Verbal IQ reflects vocabulary, knowledge, social reasoning and other “crystallized” cognitive abilities, whereas Performance IQ reflects non-verbal, more “fluid” abilities - the ability to reason and to solve novel problems. The cognitive functions assessed by Performance IQ are less dependent on culture, race and formalized learning than the “crystallized” functions evaluated on the Verbal scale. The IQ score also reflects other aspects of cognitive function such as memory, attention and problem-solving. Thus, if IQ loss is documented in children exposed to air pollution, memory, attention and problem-solving are also likely to be impaired.

Loss of cognitive functioning prevents individual children from attaining their full potential because IQ scores are highly correlated with academic performance, standardized test scores and high-school graduation rates [70, 71]. Population-wide IQ losses are also important, because reduction in the average IQ of all children in a population by as little as 2 points results in a significant decrease in the number of gifted children and a corresponding increase in the number with IQ scores below 70 [74, 75]. Any increase in the number of children with IQ scores below 70 is societally and economically significant because these children may experience a level of developmental delay that requires special education services and limits their capacity to live independently or to attain competitive employment.

Pollution-related IQ losses fall most heavily on children in Massachusetts’ most vulnerable communities, where they can magnify the impacts of poverty, racism, psychosocial stress and toxic environmental hazards such as lead [71]. The study by Wang et al. [39], one of the three studies on which we relied in developing the correlation between PM_2.5_ air pollution and Performance IQ loss, found that the inverse association between PM_2.5_ and Performance IQ was stronger among less advantaged children.

This analysis has several limitations. First, we were not able to map airborne PM_2.5_ pollution concentrations in Massachusetts as precisely as we might have wished because of the relatively small number of air pollution monitors operated by the Massachusetts Department of Environmental Protection (MassDEP). This lack of geographically fine-grained information especially hindered our ability to quantify PM_2.5_ exposure levels in minority and low-income communities. Because such communities often comprise only a portion of a city or town’s total population, their pollution levels are typically aggregated with data from the remainder of the city or town unless monitors are placed there, and thus only an average concentration can be computed. Other investigators have developed fine-grained estimates of PM_2.5_ pollution based on EPA monitoring data coupled with satellite imagery [76]. In areas of the United States with few air monitoring stations such as the Great Plains and Rocky Mountain states these models provide important, previously unavailable information. In the northeastern United States, including Massachusetts, where monitors are more numerous (though still not sufficient in number), the difference between long-term average PM_2.5_ concentration as predicted by these models and the monitored long-term average concentration is small [76].

A second limitation on this study is the lack of a published, meta-analysis–based concentration-response coefficient linking PM_2.5_ air pollution concentrations to IQ loss in children. In the absence of a published coefficient, we were forced to derive a provisional coefficient through review of the published literature and to use this coefficient to estimate air-pollution-related IQ loss in Massachusetts children. We will update this provisional estimate when a meta-analysis–based concentration-response coefficient becomes available.

We would have liked to have examined in greater detail the possibility of an interaction between PM_2.5_ pollution and social disadvantage in their effects on children’s cognitive functioning. The report by Wang et al. [39] reported effect modification of the association between PM_2.5_ and Performance IQ by socio-economic status, with the inverse correlation being stronger among less advantaged children. However, the other two studies on which we relied (Harris et al., 2015 [37]; Porta et al., 2016 [38]) did not report analyses stratified by socio-economic status, and therefore we were not able to take this heterogeneity in risk into account in our analyses.

Disease and premature death caused by air pollution can be prevented. The 74% reduction in air pollutant emissions that has been achieved in the United States since passage of the Clean Air Act in 1970 and similar reductions seen in other countries demonstrate clearly that air pollution can be controlled by laws, regulations and technologies that are based on science, backed by enforcement and encouraged by incentives [1, 50–52]. Immediate control of air pollution and prevention of air-pollution-related disease in Massachusetts and across the United States will require tightening of EPA pollution control standards to at least bring them into line with World Health Organization guidelines. In Table 4, we present examples of actions that could be taken at the local and state levels in Massachusetts as well as nationally to control air pollution and prevent pollution-related disease.

**Table 4:** Examples of recommendations to reduce air pollution and prevent pollution-related disease

**Community-level Recommendations:**	
•.Convert all municipal vehicle fleets – cars, trucks, buses - to hybrid and fully electric vehicles	
•.Place solar panels on the roofs of municipal buildings	
•.Preferentially purchase electricity produced by renewable energy	
•.Block construction of gas pipelines, compressor stations and other components of the natural gas network	
•.Prohibit gas hook-ups in new construction	
•.Revise building codes to increase energy efficiency	
**State- and National-Level Recommendations:**	
•.State authorities must urge the US Environmental Protection to tighten federal air quality standards for PM_2.5_ pollution to better protect health. The occurrence of disease, premature death and cognitive impairment at PM_2.5_ pollution levels below current federal standards is clear evidence that these standards are not sufficiently protective of health. Current federal air pollution standards fail especially to protect the health of children. A critical next step will be lowering of the federal air quality standard for PM_2.5_ pollution to at least 5 μg/M^3^.	
•.State Departments of Environmental Protection must add more air monitoring stations and increase the density of the statewide Ambient Air Quality Monitoring Network. There is particular need to prioritize placement of air monitoring stations in economically disadvantaged and socially vulnerable communities. The current sparse network of air monitoring stations makes it impossible to quantify air pollution’s health effects at the neighborhood level and thus impedes assessment of localized differences in air pollution’s health impacts.	
•.State Departments of Environmental Protection must publish an annually updated, open-source air pollution emissions inventory in an easily accessible, interactive dashboard-style format.	
•.State Departments of Public Health must create an open-access dashboard that annually tracks and publicizes information on pollution-related disease and death in each county, city and town	
•.State and national governments must require operators of electric power grids to favor renewable energy over electricity produced by fossil fuel combustion	
•.State and national governments must reduce pollutant emissions and air pollution levels by accelerating progress away from fossil fuels toward net zero carbon. Fossil fuel combustion is the major source of both air pollution and greenhouse gas emissions and is the predominant source of the air pollution produced in Massachusetts. The most effective strategy for reducing air pollution, preventing air-pollution-related disease, and achieving net zero carbon is through a rapid, wide-scale, government-supported transition from away all fossil fuels — coal, gas, and oil – to clean, renewable energy. Two powerful tools for accelerating this transition are to phase out all governmental subsidies and tax breaks for the fossil fuel industry, while at the same time increasing incentives at both the individual and system levels for wind and solar power.	
•.State and national governments must recognize the significant health and environmental impacts of methane gas and resist the temptation to continue to rely on gas for power generation and heating. Methane gas has been marketed as a “transition” fuel – a “bridge” from coal and oil to the clean energy sources of the future. However while methane provides some reductions in pollution compared to coal and oil, it is a polluting fuel, a potent driver of global warming and is associated with health and environmental hazards at every stage of its life cycle. Gas extraction by hydraulic fracturing, “fracking”, releases vast quantities of methane to the atmosphere and contaminates air and groundwater. Pipelines and compressor stations experience leaks and explosions. Gas combustion generates greenhouse gases and air pollution by oxides of nitrogen (NO_X_).	
•.State and national governments must end all subsidies and tax breaks for the fossil fuel industry	
•.State and national governments must resist any temptation to move to nuclear power	

Enduring control of air pollution will be most effectively achieved by a massive, wide-scale transition away from all fossil fuels to clean, renewable energy. Two very encouraging developments increase the likelihood that such a transition could occur within the next decade. The first is an almost 500% increase since 2010 in the fraction of electricity generated from wind and solar power, with the result that in 2021 investment in renewables surpassed all spending on oil and gas exploration for the first time [77]. The second development is steep reduction in the cost of producing electricity from renewables. The cost of generating electricity from solar energy has fallen by nearly 90% since 2010 and from wind by more than 50% [78]. These costs are projected to decline still further over the next five years as additional economies of scale are realized. It is now cheaper in many places to produce electricity from renewables than from any fossil fuel [79].

The impediments to air pollution control are no longer technical, but rather are economic and political. Key to control of air pollution and a rapid transition to clean, non-polluting energy will be courageous and visionary political leaders who heed the science, recognize pollution’s great dangers, and take bold, evidence-based action to stop pollution at its sources. Publication of community-level data on pollution’s health effects has potential to increase awareness of pollution’s dangers among policy-makers and the public and thus to catalyze preventive intervention [53–55].

## Supplementary Information

**Additional file 1.**

## Data Availability

All materials can be made fully available.
