# Supplementary material for: A replicable strategy for mapping air pollution’s community-level health impacts and catalyzing prevention
Source: Environ Health. 2022 Jul 18;21:70. doi: 10.1186/s12940-022-00879-3 (PMC9288863; doi:10.1186/s12940-022-00879-3)
Supplement: Supplementary file 1 — Additional file 1. [file 12940_2022_879_MOESM1_ESM.docx]

**Table 5. Estimated Deaths (All Causes) Attributable to PM_2.5_ Air Pollution by City and Town, Massachusetts, 2019**

| **City/**  **Town** | **Total Population** | **Estimated Number of Deaths**  **Attributable to PM_2.5_  Air Pollution** | **Deaths per 1,000 Attributable**  **to PM_2.5_ Air Pollution** |
| --- | --- | --- | --- |
|  |  |  | |
| Abington | 16,668 | 6 | 0.36 |
| Acton | 23,662 | 6 | 0.26 |
| Acushnet | 10,625 | 5 | 0.50 |
| Adams | 8,010 | 4 | 0.54 |
| Agawam | 28,613 | 19 | 0.66 |
| Alford | 488 | 0 | 0.40 |
| Amesbury | 17,532 | 6 | 0.35 |
| Amherst | 39,924 | 5 | 0.13 |
| Andover | 36,356 | 8 | 0.23 |
| Aquinnah | 320 | 0 | 0.14 |
| Arlington | 45,531 | 19 | 0.43 |
| Ashburnham | 6,348 | 3 | 0.46 |
| Ashby | 3,219 | 1 | 0.42 |
| Ashfield | 1,717 | 0 | 0.26 |
| Ashland | 17,807 | 6 | 0.33 |
| Athol | 11,732 | 6 | 0.48 |
| Attleboro | 45,237 | 20 | 0.45 |
| Auburn | 16,766 | 11 | 0.67 |
| Avon | 4,549 | 2 | 0.50 |
| Ayer | 8,196 | 5 | 0.55 |
| Barnstable | 44,477 | 24 | 0.53 |
| Barre | 5,578 | 2 | 0.30 |
| Becket | 1,716 | 1 | 0.63 |
| Bedford | 14,123 | 7 | 0.52 |
| Belchertown | 15,098 | 4 | 0.24 |
| Bellingham | 17,270 | 7 | 0.39 |
| Belmont | 26,116 | 8 | 0.32 |
| Berkley | 6,851 | 2 | 0.30 |
| Berlin | 3,240 | 2 | 0.56 |
| Bernardston | 2,090 | 1 | 0.35 |
| Beverly | 42,174 | 19 | 0.45 |
| Billerica | 43,367 | 17 | 0.39 |
| Blackstone | 9,288 | 4 | 0.41 |
| Blandford | 1,252 | 0 | 0.34 |
| Bolton | 5,426 | 1 | 0.24 |
| Boston | 692,600 | 220 | 0.32 |
| Bourne | 19,762 | 12 | 0.60 |
| Boxborough | 5,793 | 1 | 0.21 |
| Boxford | 8,332 | 2 | 0.23 |
| Boylston | 4,712 | 2 | 0.39 |
| Braintree | 37,190 | 22 | 0.58 |
| Brewster | 9,775 | 7 | 0.76 |
| Bridgewater | 27,619 | 9 | 0.31 |
| Brimfield | 3,680 | 2 | 0.48 |
| Brockton | 95,708 | 41 | 0.42 |
| Brookfield | 3,452 | 2 | 0.59 |
| Brookline | 59,121 | 17 | 0.28 |
| Buckland | 1,850 | 1 | 0.37 |
| Burlington | 28,627 | 12 | 0.41 |
| Cambridge | 118,927 | 26 | 0.22 |
| Canton | 23,805 | 12 | 0.50 |
| Carlisle | 5,252 | 1 | 0.25 |
| Carver | 11,767 | 5 | 0.44 |
| Charlemont | 1,233 | 1 | 0.52 |
| Charlton | 13,713 | 7 | 0.54 |
| Chatham | 5,982 | 5 | 0.90 |
| Chelmsford | 35,391 | 16 | 0.45 |
| Chelsea | 39,690 | 14 | 0.34 |
| Cheshire | 3,129 | 2 | 0.55 |
| Chester | 1,369 | 1 | 0.39 |
| Chesterfield | 1,249 | 0 | 0.29 |
| Chicopee | 55,126 | 19 | 0.35 |
| Chilmark | 922 | 0 | 0.39 |
| Clarksburg | 1,638 | 1 | 0.83 |
| Clinton | 14,000 | 8 | 0.56 |
| Cohasset | 8,548 | 3 | 0.39 |
| Colrain | 1,661 | 1 | 0.33 |
| Concord | 18,918 | 8 | 0.44 |
| Conway | 1,873 | 0 | 0.24 |
| Cummington | 874 | 1 | 0.63 |
| Dalton | 6,525 | 5 | 0.71 |
| Danvers | 27,549 | 16 | 0.57 |
| Dartmouth | 34,188 | 17 | 0.50 |
| Dedham | 25,219 | 19 | 0.74 |
| Deerfield | 4,991 | 2 | 0.44 |
| Dennis | 13,871 | 13 | 0.93 |
| Dighton | 7,967 | 3 | 0.35 |
| Douglas | 9,038 | 3 | 0.35 |
| Dover | 6,127 | 2 | 0.27 |
| Dracut | 31,634 | 14 | 0.43 |
| Dudley | 11,773 | 5 | 0.43 |
| Dunstable | 3,403 | 1 | 0.37 |
| Duxbury | 15,921 | 6 | 0.37 |
| East Bridgewater | 14,526 | 6 | 0.39 |
| East Brookfield | 2,210 | 1 | 0.56 |
| East Longmeadow | 16,192 | 12 | 0.73 |
| Eastham | 4,906 | 3 | 0.70 |
| Easthampton | 15,829 | 6 | 0.35 |
| Easton | 25,105 | 8 | 0.34 |
| Edgartown | 4,348 | 2 | 0.39 |
| Egremont | 1,205 | 0 | 0.32 |
| Erving | 1,750 | 1 | 0.34 |
| Essex | 3,799 | 1 | 0.35 |
| Everett | 46,451 | 15 | 0.33 |
| Fairhaven | 16,078 | 11 | 0.66 |
| Fall River | 89,541 | 48 | 0.53 |
| Falmouth | 30,993 | 20 | 0.66 |
| Fitchburg | 40,638 | 22 | 0.55 |
| Florida | 715 | 0 | 0.54 |
| Foxborough | 18,399 | 6 | 0.30 |
| Framingham | 74,416 | 27 | 0.36 |
| Franklin | 34,087 | 9 | 0.27 |
| Freetown | 9,394 | 3 | 0.36 |
| Gardner | 20,683 | 4 | 0.18 |
| Georgetown | 8,768 | 2 | 0.23 |
| Gill | 1,465 | 0 | 0.31 |
| Gloucester | 30,430 | 15 | 0.48 |
| Goshen | 1,059 | 0 | 0.13 |
| Gosnold | 75 | 0 | 0.61 |
| Grafton | 18,883 | 7 | 0.35 |
| Granby | 6,291 | 1 | 0.22 |
| Granville | 1,611 | 1 | 0.46 |
| Great Barrington | 6,945 | 5 | 0.75 |
| Greenfield | 17,258 | 10 | 0.60 |
| Groton | 11,325 | 3 | 0.28 |
| Groveland | 6,849 | 2 | 0.29 |
| Hadley | 5,342 | 2 | 0.38 |
| Halifax | 7,896 | 3 | 0.38 |
| Hamilton | 8,051 | 2 | 0.25 |
| Hampden | 5,177 | 4 | 0.69 |
| Hancock | 696 | 0 | 0.42 |
| Hanover | 14,570 | 4 | 0.27 |
| Hanson | 10,914 | 4 | 0.35 |
| Hardwick | 3,057 | 1 | 0.29 |
| Harvard | 6,620 | 2 | 0.28 |
| Harwich | 12,142 | 9 | 0.76 |
| Hatfield | 3,251 | 1 | 0.34 |
| Haverhill | 64,014 | 24 | 0.38 |
| Hawley | 334 | 0 | 0.51 |
| Heath | 695 | 0 | 0.52 |
| Hingham | 24,679 | 14 | 0.58 |
| Hinsdale | 1,911 | 0 | 0.20 |
| Holbrook | 11,033 | 5 | 0.50 |
| Holden | 19,303 | 7 | 0.35 |
| Holland | 2,482 | 1 | 0.34 |
| Holliston | 14,912 | 4 | 0.29 |
| Holyoke | 40,117 | 16 | 0.40 |
| Hopedale | 5,951 | 3 | 0.56 |
| Hopkinton | 18,470 | 5 | 0.28 |
| Hubbardston | 4,829 | 2 | 0.36 |
| Hudson | 19,864 | 10 | 0.48 |
| Hull | 10,475 | 5 | 0.49 |
| Huntington | 2,169 | 1 | 0.31 |
| Ipswich | 14,074 | 6 | 0.42 |
| Kingston | 13,863 | 6 | 0.47 |
| Lakeville | 11,561 | 5 | 0.40 |
| Lancaster | 8,082 | 3 | 0.42 |
| Lanesborough | 2,940 | 2 | 0.56 |
| Lawrence | 80,028 | 20 | 0.25 |
| Lee | 5,664 | 4 | 0.76 |
| Leicester | 11,341 | 6 | 0.50 |
| Lenox | 4,944 | 5 | 0.97 |
| Leominster | 41,716 | 26 | 0.62 |
| Leverett | 1,837 | 1 | 0.32 |
| Lexington | 33,132 | 13 | 0.39 |
| Leyden | 715 | 0 | 0.32 |
| Lincoln | 7,052 | 3 | 0.39 |
| Littleton | 10,227 | 4 | 0.37 |
| Longmeadow | 15,705 | 9 | 0.59 |
| Lowell | 110,997 | 41 | 0.37 |
| Ludlow | 21,233 | 7 | 0.32 |
| Lunenburg | 11,736 | 6 | 0.49 |
| Lynn | 94,299 | 34 | 0.36 |
| Lynnfield | 12,999 | 5 | 0.35 |
| Malden | 60,470 | 19 | 0.32 |
| Manchester-by-the-Sea | 5,434 | 2 | 0.30 |
| Mansfield | 24,470 | 7 | 0.28 |
| Marblehead | 20,555 | 7 | 0.34 |
| Marion | 5,188 | 3 | 0.53 |
| Marlborough | 39,597 | 18 | 0.46 |
| Marshfield | 25,967 | 11 | 0.43 |
| Mashpee | 14,229 | 8 | 0.53 |
| Mattapoisett | 6,401 | 3 | 0.49 |
| Maynard | 11,336 | 4 | 0.33 |
| Medfield | 12,955 | 4 | 0.30 |
| Medford | 57,341 | 29 | 0.50 |
| Medway | 13,479 | 4 | 0.33 |
| Melrose | 28,016 | 9 | 0.32 |
| Mendon | 6,223 | 2 | 0.29 |
| Merrimac | 6,960 | 1 | 0.20 |
| Methuen | 50,706 | 19 | 0.37 |
| Middleborough | 25,463 | 12 | 0.48 |
| Middlefield | 534 | 0 | 0.46 |
| Middleton | 10,110 | 4 | 0.35 |
| Milford | 29,101 | 15 | 0.50 |
| Millbury | 13,947 | 7 | 0.53 |
| Millis | 8,310 | 3 | 0.38 |
| Millville | 3,257 | 1 | 0.36 |
| Milton | 27,593 | 14 | 0.50 |
| Monroe | 115 | 0 | - |
| Monson | 8,787 | 2 | 0.27 |
| Montague | 8,212 | 5 | 0.63 |
| Monterey | 924 | 0 | 0.32 |
| Montgomery | 866 | 0 | 0.43 |
| Mount Washington | 157 | 0 | 0.31 |
| Nahant | 3,513 | 2 | 0.48 |
| Nantucket | 11,399 | 3 | 0.29 |
| Natick | 36,050 | 15 | 0.41 |
| Needham | 31,388 | 15 | 0.48 |
| New Ashford | 223 | 0 | 0.38 |
| New Bedford | 95,363 | 46 | 0.49 |
| New Braintree | 1,024 | 0 | 0.15 |
| New Marlborough | 1,458 | 1 | 0.54 |
| New Salem | 1,021 | 0 | 0.19 |
| Newbury | 7,148 | 2 | 0.28 |
| Newburyport | 18,289 | 8 | 0.42 |
| Newton | 88,414 | 34 | 0.38 |
| Norfolk | 12,003 | 3 | 0.24 |
| North Adams | 12,730 | 8 | 0.64 |
| North Andover | 31,188 | 11 | 0.35 |
| North Attleborough | 29,364 | 10 | 0.32 |
| North Brookfield | 4,792 | 2 | 0.36 |
| North Reading | 15,865 | 5 | 0.33 |
| Northampton | 28,451 | 8 | 0.29 |
| Northborough | 15,109 | 7 | 0.43 |
| Northbridge | 16,679 | 9 | 0.55 |
| Northfield | 2,958 | 1 | 0.38 |
| Norton | 19,948 | 7 | 0.36 |
| Norwell | 11,153 | 4 | 0.36 |
| Norwood | 29,725 | 18 | 0.61 |
| Oak Bluffs | 4,667 | 2 | 0.42 |
| Oakham | 1,957 | 1 | 0.29 |
| Orange | 7,582 | 4 | 0.48 |
| Orleans | 5,788 | 5 | 0.79 |
| Otis | 1,539 | 1 | 0.35 |
| Oxford | 14,009 | 7 | 0.49 |
| Palmer | 12,232 | 5 | 0.40 |
| Paxton | 4,963 | 2 | 0.37 |
| Peabody | 53,070 | 35 | 0.66 |
| Pelham | 1,313 | 0 | 0.27 |
| Pembroke | 18,509 | 7 | 0.36 |
| Pepperell | 12,114 | 4 | 0.30 |
| Peru | 834 | 1 | 1.05 |
| Petersham | 1,250 | 0 | 0.34 |
| Phillipston | 1,746 | 0 | 0.20 |
| Pittsfield | 42,142 | 27 | 0.64 |
| Plainfield | 661 | 0 | 0.39 |
| Plainville | 9,293 | 4 | 0.41 |
| Plymouth | 61,528 | 25 | 0.41 |
| Plympton | 2,987 | 1 | 0.39 |
| Princeton | 3,488 | 1 | 0.27 |
| Provincetown | 2,961 | 2 | 0.58 |
| Quincy | 94,470 | 44 | 0.47 |
| Randolph | 34,362 | 13 | 0.38 |
| Raynham | 14,470 | 7 | 0.51 |
| Reading | 25,400 | 9 | 0.35 |
| Rehoboth | 12,385 | 3 | 0.27 |
| Revere | 53,073 | 21 | 0.40 |
| Richmond | 1,416 | 0 | 0.28 |
| Rochester | 5,687 | 1 | 0.23 |
| Rockland | 17,986 | 9 | 0.48 |
| Rockport | 7,282 | 4 | 0.53 |
| Rowe | 389 | 0 | 0.33 |
| Rowley | 6,473 | 2 | 0.32 |
| Royalston | 1,277 | 0 | 0.24 |
| Russell | 1,792 | 1 | 0.47 |
| Rutland | 8,938 | 3 | 0.37 |
| Salem | 43,226 | 16 | 0.36 |
| Salisbury | 9,534 | 3 | 0.34 |
| Sandisfield | 891 | 0 | 0.44 |
| Sandwich | 20,169 | 9 | 0.47 |
| Saugus | 28,361 | 14 | 0.48 |
| Savoy | 675 | 0 | 0.32 |
| Scituate | 18,924 | 8 | 0.40 |
| Seekonk | 15,770 | 6 | 0.35 |
| Sharon | 18,895 | 4 | 0.23 |
| Sheffield | 3,129 | 2 | 0.53 |
| Shelburne | 1,837 | 1 | 0.29 |
| Sherborn | 4,335 | 1 | 0.18 |
| Shirley | 7,636 | 4 | 0.46 |
| Shrewsbury | 38,526 | 16 | 0.42 |
| Shutesbury | 1,754 | 0 | 0.22 |
| Somerset | 18,129 | 11 | 0.62 |
| Somerville | 81,360 | 23 | 0.28 |
| South Hadley | 17,625 | 6 | 0.34 |
| Southampton | 6,171 | 1 | 0.24 |
| Southborough | 10,208 | 3 | 0.26 |
| Southbridge | 16,878 | 11 | 0.67 |
| Southwick | 9,740 | 5 | 0.49 |
| Spencer | 11,935 | 6 | 0.53 |
| Springfield | 153,606 | 69 | 0.45 |
| Sterling | 8,174 | 4 | 0.50 |
| Stockbridge | 1,890 | 1 | 0.72 |
| Stoneham | 24,126 | 11 | 0.45 |
| Stoughton | 28,915 | 12 | 0.42 |
| Stow | 7,234 | 2 | 0.26 |
| Sturbridge | 9,597 | 4 | 0.37 |
| Sudbury | 19,655 | 5 | 0.28 |
| Sunderland | 3,629 | 1 | 0.34 |
| Sutton | 9,582 | 3 | 0.35 |
| Swampscott | 15,298 | 5 | 0.36 |
| Swansea | 16,834 | 9 | 0.51 |
| Taunton | 57,464 | 27 | 0.48 |
| Templeton | 8,138 | 3 | 0.35 |
| Tewksbury | 31,178 | 14 | 0.45 |
| Tisbury | 4,096 | 1 | 0.37 |
| Tolland | 508 | 0 | 0.48 |
| Topsfield | 6,641 | 3 | 0.45 |
| Townsend | 9,506 | 4 | 0.39 |
| Truro | 2,008 | 1 | 0.70 |
| Tyngsborough | 12,527 | 3 | 0.24 |
| Tyringham | 312 | 0 | 0.47 |
| Upton | 8,065 | 2 | 0.31 |
| Uxbridge | 14,195 | 6 | 0.45 |
| Wakefield | 27,045 | 11 | 0.41 |
| Wales | 1,874 | 1 | 0.43 |
| Walpole | 25,200 | 11 | 0.44 |
| Waltham | 62,495 | 24 | 0.39 |
| Ware | 9,711 | 5 | 0.47 |
| Wareham | 22,745 | 13 | 0.55 |
| Warren | 5,222 | 2 | 0.31 |
| Warwick | 769 | 1 | 0.70 |
| Washington | 541 | 0 | 0.18 |
| Watertown | 35,939 | 15 | 0.42 |
| Wayland | 13,835 | 1 | 0.04 |
| Webster | 16,949 | 14 | 0.80 |
| Wellesley | 28,670 | 9 | 0.31 |
| Wellfleet | 2,724 | 1 | 0.45 |
| Wendell | 878 | 0 | 0.21 |
| Wenham | 5,278 | 2 | 0.29 |
| West Boylston | 8,077 | 7 | 0.82 |
| West Bridgewater | 7,281 | 4 | 0.50 |
| West Brookfield | 3,727 | 2 | 0.48 |
| West Newbury | 4,714 | 1 | 0.24 |
| West Springfield | 28,517 | 17 | 0.59 |
| West Stockbridge | 1,257 | 1 | 0.47 |
| West Tisbury | 2,904 | 1 | 0.30 |
| Westborough | 19,144 | 9 | 0.49 |
| Westfield | 41,204 | 21 | 0.50 |
| Westford | 24,817 | 6 | 0.25 |
| Westhampton | 1,637 | 0 | 0.23 |
| Westminster | 7,997 | 3 | 0.42 |
| Weston | 12,124 | 7 | 0.57 |
| Westport | 16,034 | 8 | 0.50 |
| Westwood | 16,400 | 8 | 0.48 |
| Weymouth | 57,746 | 28 | 0.49 |
| Whately | 1,567 | 1 | 0.55 |
| Whitman | 15,216 | 5 | 0.34 |
| Wilbraham | 14,689 | 5 | 0.32 |
| Williamsburg | 2,466 | 1 | 0.49 |
| Williamstown | 7,434 | 3 | 0.42 |
| Wilmington | 23,445 | 8 | 0.36 |
| Winchendon | 10,905 | 3 | 0.32 |
| Winchester | 22,799 | 9 | 0.41 |
| Windsor | 866 | 0 | 0.34 |
| Winthrop | 18,544 | 9 | 0.51 |
| Woburn | 40,228 | 23 | 0.56 |
| Worcester | 185,428 | 93 | 0.50 |
| Worthington | 1,175 | 0 | 0.37 |
| Wrentham | 12,023 | 6 | 0.47 |
| Yarmouth | 23,203 | 19 | 0.82 |
| **TOTAL** |  | **2,780** |  |

**Table 6. Estimated Adult Deaths and Pediatric Disease Cases Attributable to PM_2.5_ Air Pollution by Cause by City and Town, Massachusetts, 2019**

| **Town** | **Low Birth Weight Infants** | **Pediatric Asthma Cases** | **Heart Disease**  **Deaths** | **Lung Cancer**  **Deaths** | **Stroke**  **Deaths** | **Chronic Lower Respiratory Disease (CLRD)**  **Deaths** |
| --- | --- | --- | --- | --- | --- | --- |
| Abington | 1 | 26 | 4 | 6 | 0 | 0 |
| Acton | 1 | 80 | 3 | 4 | 0 | 1 |
| Acushnet | 0 | 21 | 3 | 4 | 0 | 0 |
| Adams | 0 | 19 | 3 | 3 | 1 | 1 |
| Agawam | 1 | 64 | 12 | 15 | 2 | 2 |
| Alford | 0 | 0 | 0 | 0 | 0 | 0 |
| Amesbury | 0 | 16 | 4 | 5 | 0 | 1 |
| Amherst | 1 | 26 | 3 | 2 | 0 | 1 |
| Andover | 0 | 79 | 6 | 6 | 1 | 1 |
| Aquinnah | 0 | 0 | 0 | 0 | 0 | 0 |
| Arlington | 3 | 85 | 11 | 15 | 1 | 2 |
| Ashburnham | 0 | 18 | 2 | 1 | 0 | 0 |
| Ashby | 0 | 11 | 1 | 1 | 0 | 1 |
| Ashfield | 0 | 3 | 0 | 0 | 0 | 0 |
| Ashland | 1 | 41 | 3 | 6 | 0 | 1 |
| Athol | 0 | 31 | 4 | 5 | 0 | 1 |
| Attleboro | 2 | 73 | 10 | 15 | 1 | 3 |
| Auburn | 1 | 40 | 7 | 8 | 1 | 1 |
| Avon | 0 | 10 | 2 | 2 | 0 | 0 |
| Ayer | 1 | 12 | 3 | 3 | 0 | 1 |
| Barnstable | 2 | 58 | 16 | 17 | 2 | 3 |
| Barre | 0 | 6 | 1 | 1 | 0 | 0 |
| Becket | 0 | 2 | 1 | 1 | 0 | 0 |
| Bedford | 0 | 20 | 3 | 4 | 0 | 1 |
| Belchertown | 0 | 23 | 2 | 2 | 0 | 1 |
| Bellingham | 0 | 15 | 6 | 6 | 0 | 1 |
| Belmont | 1 | 74 | 4 | 8 | 0 | 1 |
| Berkley | 0 | 11 | 1 | 1 | 0 | 0 |
| Berlin | 0 | 4 | 1 | 1 | 0 | 0 |
| Bernardston | 0 | 5 | 1 | 1 | 0 | 0 |
| Beverly | 1 | 67 | 15 | 14 | 2 | 2 |
| Billerica | 3 | 59 | 10 | 17 | 1 | 3 |
| Blackstone | 0 | 11 | 3 | 3 | 0 | 0 |
| Blandford | 0 | 2 | 0 | 1 | 0 | 0 |
| Bolton | 0 | 8 | 1 | 1 | 0 | 0 |
| Boston | 47 | 1840 | 121 | 176 | 15 | 21 |
| Bourne | 1 | 23 | 6 | 9 | 1 | 2 |
| Boxborough | 0 | 4 | 0 | 1 | 0 | 0 |
| Boxford | 0 | 11 | 1 | 2 | 0 | 0 |
| Boylston | 0 | 8 | 1 | 1 | 0 | 0 |
| Braintree | 2 | 107 | 13 | 15 | 1 | 3 |
| Brewster | 0 | 14 | 6 | 5 | 1 | 0 |
| Bridgewater | 0 | 39 | 7 | 8 | 0 | 1 |
| Brimfield | 0 | 7 | 0 | 1 | 0 | 0 |
| Brockton | 9 | 366 | 25 | 33 | 2 | 3 |
| Brookfield | 0 | 6 | 1 | 2 | 0 | 0 |
| Brookline | 3 | 91 | 12 | 12 | 1 | 1 |
| Buckland | 0 | 1 | 1 | 1 | 0 | 0 |
| Burlington | 1 | 61 | 7 | 9 | 1 | 2 |
| Cambridge | 4 | 139 | 15 | 21 | 2 | 3 |
| Canton | 1 | 61 | 8 | 9 | 1 | 1 |
| Carlisle | 0 | 13 | 1 | 1 | 0 | 0 |
| Carver | 1 | 18 | 3 | 7 | 0 | 0 |
| Charlemont | 0 | 4 | 1 | 0 | 0 | 0 |
| Charlton | 1 | 30 | 5 | 4 | 1 | 1 |
| Chatham | 0 | 4 | 7 | 4 | 0 | 1 |
| Chelmsford | 1 | 104 | 9 | 11 | 1 | 2 |
| Chelsea | 4 | 111 | 7 | 11 | 1 | 2 |
| Cheshire | 0 | 5 | 0 | 2 | 0 | 0 |
| Chester | 0 | 0 | 0 | 1 | 0 | 0 |
| Chesterfield | 0 | 3 | 0 | 0 | 0 | 0 |
| Chicopee | 2 | 128 | 11 | 16 | 1 | 3 |
| Chilmark | 0 | 0 | 0 | 0 | 0 | 0 |
| Clarksburg | 0 | 2 | 1 | 1 | 0 | 0 |
| Clinton | 2 | 24 | 6 | 6 | 1 | 1 |
| Cohasset | 0 | 14 | 2 | 2 | 0 | 0 |
| Colrain | 0 | 1 | 1 | 1 | 0 | 0 |
| Concord | 0 | 41 | 3 | 4 | 1 | 1 |
| Conway | 0 | 3 | 0 | 0 | 0 | 0 |
| Cummington | 0 | 0 | 0 | 0 | 0 | 0 |
| Dalton | 0 | 15 | 3 | 3 | 0 | 0 |
| Danvers | 1 | 44 | 9 | 11 | 2 | 1 |
| Dartmouth | 1 | 85 | 10 | 10 | 1 | 1 |
| Dedham | 1 | 54 | 11 | 13 | 1 | 1 |
| Deerfield | 0 | 8 | 1 | 1 | 0 | 0 |
| Dennis | 0 | 12 | 7 | 9 | 0 | 2 |
| Dighton | 0 | 13 | 2 | 2 | 0 | 0 |
| Douglas | 0 | 35 | 3 | 3 | 0 | 0 |
| Dover | 0 | 16 | 1 | 1 | 0 | 0 |
| Dracut | 2 | 107 | 8 | 14 | 0 | 1 |
| Dudley | 0 | 27 | 4 | 5 | 0 | 1 |
| Dunstable | 0 | 9 | 1 | 1 | 0 | 0 |
| Duxbury | 0 | 37 | 5 | 4 | 0 | 1 |
| East Bridgewater | 0 | 20 | 4 | 4 | 0 | 1 |
| East Brookfield | 0 | 5 | 1 | 1 | 0 | 0 |
| East Longmeadow | 1 | 60 | 6 | 8 | 1 | 2 |
| Eastham | 0 | 6 | 3 | 4 | 0 | 0 |
| Easthampton | 0 | 15 | 4 | 4 | 0 | 1 |
| Easton | 1 | 63 | 4 | 7 | 0 | 1 |
| Edgartown | 0 | 5 | 1 | 2 | 0 | 0 |
| Egremont | 0 | 0 | 0 | 0 | 0 | 0 |
| Erving | 0 | 3 | 1 | 1 | 0 | 0 |
| Essex | 0 | 5 | 1 | 2 | 0 | 0 |
| Everett | 4 | 105 | 10 | 13 | 1 | 2 |
| Fairhaven | 0 | 31 | 6 | 6 | 1 | 1 |
| Fall River | 6 | 321 | 28 | 38 | 3 | 6 |
| Falmouth | 1 | 71 | 13 | 16 | 2 | 1 |
| Fitchburg | 3 | 160 | 13 | 17 | 3 | 3 |
| Florida | 0 | 0 | 0 | 0 | 0 | 0 |
| Foxborough | 1 | 51 | 3 | 6 | 0 | 1 |
| Framingham | 4 | 156 | 18 | 20 | 2 | 5 |
| Franklin | 1 | 66 | 5 | 6 | 0 | 1 |
| Freetown | 0 | 13 | 3 | 3 | 0 | 0 |
| Gardner | 0 | 73 | 8 | 12 | 3 | 1 |
| Georgetown | 0 | 12 | 2 | 1 | 0 | 0 |
| Gill | 0 | 1 | 1 | 1 | 0 | 0 |
| Gloucester | 1 | 37 | 8 | 15 | 1 | 2 |
| Goshen | 0 | 3 | 0 | 0 | 0 | 0 |
| Gosnold | 0 | 0 | 0 | 0 | 0 | 0 |
| Grafton | 1 | 56 | 4 | 6 | 0 | 1 |
| Granby | 0 | 12 | 1 | 2 | 0 | 0 |
| Granville | 0 | 1 | 1 | 1 | 0 | 0 |
| Great Barrington | 0 | 17 | 2 | 3 | 0 | 0 |
| Greenfield | 0 | 32 | 6 | 7 | 1 | 2 |
| Groton | 1 | 35 | 2 | 2 | 0 | 0 |
| Groveland | 0 | 11 | 1 | 2 | 0 | 0 |
| Hadley | 0 | 8 | 1 | 1 | 0 | 0 |
| Halifax | 0 | 9 | 2 | 3 | 0 | 0 |
| Hamilton | 0 | 10 | 2 | 2 | 0 | 0 |
| Hampden | 0 | 13 | 2 | 1 | 0 | 0 |
| Hancock | 0 | 0 | 0 | 0 | 0 | 0 |
| Hanover | 0 | 27 | 3 | 4 | 0 | 0 |
| Hanson | 0 | 21 | 2 | 4 | 0 | 0 |
| Hardwick | 0 | 3 | 0 | 1 | 0 | 0 |
| Harvard | 0 | 9 | 0 | 1 | 0 | 0 |
| Harwich | 0 | 12 | 7 | 8 | 1 | 1 |
| Hatfield | 0 | 4 | 0 | 1 | 0 | 0 |
| Haverhill | 3 | 154 | 16 | 18 | 1 | 3 |
| Hawley | 0 | 0 | 0 | 0 | 0 | 0 |
| Heath | 0 | 0 | 0 | 0 | 0 | 0 |
| Hingham | 1 | 58 | 8 | 6 | 1 | 1 |
| Hinsdale | 0 | 2 | 2 | 1 | 0 | 0 |
| Holbrook | 1 | 26 | 4 | 4 | 0 | 1 |
| Holden | 1 | 49 | 5 | 6 | 0 | 1 |
| Holland | 0 | 8 | 1 | 1 | 0 | 0 |
| Holliston | 0 | 33 | 3 | 4 | 0 | 0 |
| Holyoke | 2 | 112 | 8 | 8 | 1 | 2 |
| Hopedale | 0 | 16 | 2 | 3 | 0 | 0 |
| Hopkinton | 1 | 65 | 4 | 4 | 0 | 0 |
| Hubbardston | 0 | 4 | 1 | 2 | 0 | 0 |
| Hudson | 1 | 67 | 6 | 7 | 1 | 0 |
| Hull | 0 | 11 | 3 | 5 | 0 | 1 |
| Huntington | 0 | 2 | 0 | 1 | 0 | 0 |
| Ipswich | 0 | 20 | 4 | 4 | 0 | 0 |
| Kingston | 0 | 25 | 4 | 5 | 0 | 1 |
| Lakeville | 0 | 20 | 3 | 3 | 0 | 1 |
| Lancaster | 0 | 16 | 0 | 2 | 0 | 0 |
| Lanesborough | 0 | 5 | 1 | 1 | 0 | 0 |
| Lawrence | 6 | 304 | 10 | 14 | 1 | 2 |
| Lee | 0 | 9 | 2 | 4 | 0 | 1 |
| Leicester | 0 | 15 | 3 | 3 | 0 | 2 |
| Lenox | 0 | 7 | 3 | 2 | 0 | 1 |
| Leominster | 2 | 123 | 12 | 17 | 2 | 4 |
| Leverett | 0 | 5 | 1 | 1 | 0 | 0 |
| Lexington | 1 | 73 | 7 | 6 | 1 | 1 |
| Leyden | 0 | 0 | 0 | 0 | 0 | 0 |
| Lincoln | 0 | 13 | 1 | 1 | 0 | 0 |
| Littleton | 0 | 23 | 1 | 3 | 0 | 0 |
| Longmeadow | 0 | 34 | 5 | 5 | 1 | 1 |
| Lowell | 8 | 379 | 21 | 32 | 2 | 6 |
| Ludlow | 0 | 30 | 5 | 4 | 1 | 1 |
| Lunenburg | 0 | 32 | 4 | 4 | 1 | 1 |
| Lynn | 7 | 269 | 20 | 31 | 3 | 5 |
| Lynnfield | 0 | 32 | 3 | 3 | 0 | 1 |
| Malden | 4 | 105 | 11 | 18 | 1 | 3 |
| Manchester-by-the-Sea | 0 | 11 | 1 | 1 | 0 | 0 |
| Mansfield | 1 | 80 | 5 | 6 | 0 | 1 |
| Marblehead | 0 | 34 | 4 | 6 | 1 | 1 |
| Marion | 0 | 13 | 1 | 2 | 0 | 0 |
| Marlborough | 3 | 117 | 15 | 13 | 1 | 2 |
| Marshfield | 1 | 48 | 5 | 10 | 0 | 1 |
| Mashpee | 0 | 31 | 5 | 8 | 1 | 1 |
| Mattapoisett | 0 | 8 | 1 | 2 | 0 | 1 |
| Maynard | 0 | 22 | 2 | 3 | 1 | 0 |
| Medfield | 0 | 53 | 2 | 3 | 0 | 0 |
| Medford | 3 | 75 | 15 | 25 | 2 | 3 |
| Medway | 1 | 40 | 4 | 4 | 0 | 1 |
| Melrose | 1 | 53 | 7 | 8 | 1 | 1 |
| Mendon | 0 | 14 | 1 | 2 | 0 | 0 |
| Merrimac | 0 | 7 | 1 | 3 | 0 | 0 |
| Methuen | 2 | 63 | 11 | 17 | 2 | 2 |
| Middleborough | 1 | 52 | 6 | 13 | 1 | 2 |
| Middlefield | 0 | 0 | 0 | 0 | 0 | 0 |
| Middleton | 0 | 18 | 2 | 3 | 0 | 1 |
| Milford | 2 | 41 | 12 | 12 | 1 | 2 |
| Millbury | 0 | 23 | 5 | 8 | 1 | 2 |
| Millis | 0 | 27 | 1 | 3 | 0 | 0 |
| Millville | 0 | 5 | 1 | 2 | 0 | 0 |
| Milton | 1 | 90 | 8 | 9 | 1 | 1 |
| Monroe | 0 | 0 | 0 | 0 | 0 | 0 |
| Monson | 0 | 12 | 2 | 2 | 0 | 0 |
| Montague | 1 | 9 | 2 | 3 | 0 | 1 |
| Monterey | 0 | 0 | 0 | 0 | 0 | 0 |
| Montgomery | 0 | 0 | 0 | 0 | 0 | 0 |
| Mount Washington | 0 | 0 | 0 | 0 | 0 | 0 |
| Nahant | 0 | 4 | 2 | 2 | 0 | 0 |
| Nantucket | 1 | 19 | 2 | 3 | 0 | 0 |
| Natick | 1 | 79 | 9 | 11 | 2 | 1 |
| Needham | 1 | 68 | 9 | 8 | 1 | 1 |
| New Ashford | 0 | 0 | 0 | 0 | 0 | 0 |
| New Bedford | 6 | 378 | 29 | 38 | 4 | 5 |
| New Braintree | 0 | 1 | 0 | 0 | 0 | 0 |
| New Marlborough | 0 | 0 | 0 | 0 | 0 | 0 |
| New Salem | 0 | 1 | 0 | 0 | 0 | 0 |
| Newbury | 0 | 7 | 2 | 2 | 0 | 0 |
| Newburyport | 0 | 28 | 4 | 4 | 0 | 1 |
| Newton | 4 | 238 | 22 | 26 | 2 | 3 |
| Norfolk | 1 | 16 | 2 | 2 | 0 | 1 |
| North Adams | 0 | 29 | 5 | 5 | 1 | 2 |
| North Andover | 0 | 52 | 8 | 6 | 1 | 1 |
| North Attleborough | 1 | 84 | 7 | 7 | 1 | 2 |
| North Brookfield | 0 | 7 | 1 | 1 | 0 | 0 |
| North Reading | 1 | 29 | 4 | 3 | 0 | 0 |
| Northampton | 0 | 51 | 6 | 5 | 1 | 1 |
| Northborough | 1 | 30 | 4 | 4 | 0 | 2 |
| Northbridge | 1 | 27 | 6 | 6 | 1 | 2 |
| Northfield | 0 | 6 | 1 | 1 | 0 | 0 |
| Norton | 1 | 17 | 4 | 5 | 0 | 1 |
| Norwell | 0 | 20 | 2 | 2 | 0 | 0 |
| Norwood | 2 | 74 | 11 | 15 | 1 | 2 |
| Oak Bluffs | 0 | 6 | 2 | 2 | 0 | 1 |
| Oakham | 0 | 2 | 0 | 1 | 0 | 0 |
| Orange | 0 | 20 | 2 | 3 | 0 | 1 |
| Orleans | 0 | 3 | 6 | 2 | 1 | 0 |
| Otis | 0 | 3 | 0 | 1 | 0 | 0 |
| Oxford | 1 | 28 | 3 | 6 | 0 | 2 |
| Palmer | 0 | 17 | 3 | 3 | 1 | 1 |
| Paxton | 0 | 4 | 1 | 1 | 0 | 0 |
| Peabody | 2 | 50 | 20 | 23 | 2 | 4 |
| Pelham | 0 | 2 | 0 | 0 | 0 | 0 |
| Pembroke | 1 | 43 | 3 | 7 | 1 | 0 |
| Pepperell | 0 | 28 | 3 | 4 | 0 | 0 |
| Peru | 0 | 0 | 0 | 0 | 0 | 0 |
| Petersham | 0 | 1 | 0 | 0 | 0 | 0 |
| Phillipston | 0 | 3 | 0 | 1 | 0 | 0 |
| Pittsfield | 3 | 118 | 15 | 22 | 3 | 5 |
| Plainfield | 0 | 0 | 0 | 0 | 0 | 0 |
| Plainville | 0 | 22 | 2 | 3 | 0 | 0 |
| Plymouth | 2 | 111 | 14 | 23 | 2 | 4 |
| Plympton | 0 | 5 | 0 | 1 | 0 | 0 |
| Princeton | 0 | 4 | 0 | 0 | 0 | 0 |
| Provincetown | 0 | 0 | 1 | 2 | 0 | 0 |
| Quincy | 6 | 119 | 29 | 45 | 4 | 8 |
| Randolph | 2 | 92 | 8 | 8 | 1 | 2 |
| Raynham | 0 | 14 | 4 | 4 | 1 | 1 |
| Reading | 1 | 45 | 5 | 6 | 1 | 1 |
| Rehoboth | 0 | 9 | 2 | 3 | 0 | 0 |
| Revere | 4 | 86 | 14 | 23 | 1 | 2 |
| Richmond | 0 | 1 | 1 | 0 | 0 | 0 |
| Rochester | 0 | 13 | 1 | 1 | 0 | 0 |
| Rockland | 1 | 36 | 4 | 7 | 0 | 1 |
| Rockport | 0 | 11 | 3 | 3 | 1 | 0 |
| Rowe | 0 | 1 | 0 | 0 | 0 | 0 |
| Rowley | 0 | 10 | 1 | 1 | 0 | 1 |
| Royalston | 0 | 2 | 0 | 0 | 0 | 0 |
| Russell | 0 | 3 | 1 | 1 | 0 | 0 |
| Rutland | 0 | 22 | 1 | 3 | 0 | 0 |
| Salem | 2 | 77 | 10 | 14 | 1 | 2 |
| Salisbury | 0 | 7 | 3 | 4 | 0 | 1 |
| Sandisfield | 0 | 0 | 0 | 0 | 0 | 0 |
| Sandwich | 1 | 39 | 3 | 7 | 1 | 1 |
| Saugus | 1 | 51 | 6 | 13 | 2 | 2 |
| Savoy | 0 | 0 | 0 | 0 | 0 | 0 |
| Scituate | 1 | 41 | 7 | 7 | 1 | 1 |
| Seekonk | 1 | 30 | 3 | 4 | 1 | 0 |
| Sharon | 1 | 52 | 4 | 5 | 0 | 0 |
| Sheffield | 0 | 4 | 1 | 2 | 0 | 0 |
| Shelburne | 0 | 4 | 1 | 0 | 0 | 0 |
| Sherborn | 0 | 16 | 1 | 1 | 0 | 0 |
| Shirley | 0 | 10 | 2 | 2 | 0 | 0 |
| Shrewsbury | 2 | 86 | 11 | 12 | 1 | 2 |
| Shutesbury | 0 | 2 | 0 | 0 | 0 | 0 |
| Somerset | 1 | 44 | 8 | 8 | 0 | 1 |
| Somerville | 3 | 93 | 14 | 22 | 2 | 2 |
| South Hadley | 0 | 25 | 4 | 4 | 1 | 1 |
| Southampton | 0 | 11 | 1 | 1 | 0 | 0 |
| Southborough | 0 | 25 | 2 | 3 | 0 | 1 |
| Southbridge | 1 | 107 | 7 | 8 | 1 | 1 |
| Southwick | 0 | 31 | 2 | 3 | 0 | 1 |
| Spencer | 1 | 28 | 4 | 7 | 0 | 1 |
| Springfield | 13 | 722 | 39 | 47 | 5 | 8 |
| Sterling | 0 | 19 | 2 | 3 | 0 | 1 |
| Stockbridge | 0 | 0 | 1 | 0 | 0 | 0 |
| Stoneham | 1 | 34 | 6 | 8 | 1 | 2 |
| Stoughton | 2 | 75 | 9 | 10 | 0 | 1 |
| Stow | 0 | 10 | 1 | 1 | 0 | 0 |
| Sturbridge | 0 | 13 | 3 | 2 | 0 | 0 |
| Sudbury | 0 | 46 | 4 | 4 | 0 | 0 |
| Sunderland | 0 | 7 | 0 | 0 | 0 | 0 |
| Sutton | 0 | 30 | 1 | 3 | 0 | 1 |
| Swampscott | 1 | 17 | 3 | 5 | 0 | 0 |
| Swansea | 0 | 46 | 3 | 7 | 0 | 1 |
| Taunton | 3 | 157 | 17 | 22 | 2 | 4 |
| Templeton | 0 | 18 | 1 | 3 | 0 | 0 |
| Tewksbury | 1 | 60 | 8 | 13 | 1 | 2 |
| Tisbury | 0 | 9 | 1 | 1 | 0 | 0 |
| Tolland | 0 | 0 | 0 | 0 | 0 | 0 |
| Topsfield | 0 | 11 | 1 | 2 | 0 | 1 |
| Townsend | 0 | 30 | 1 | 3 | 0 | 0 |
| Truro | 0 | 2 | 1 | 1 | 0 | 0 |
| Tyngsborough | 0 | 31 | 2 | 4 | 0 | 1 |
| Tyringham | 0 | 0 | 0 | 0 | 0 | 0 |
| Upton | 0 | 20 | 1 | 3 | 0 | 0 |
| Uxbridge | 1 | 32 | 4 | 6 | 0 | 0 |
| Wakefield | 1 | 39 | 7 | 8 | 0 | 2 |
| Wales | 0 | 6 | 0 | 0 | 0 | 0 |
| Walpole | 1 | 79 | 7 | 10 | 1 | 2 |
| Waltham | 4 | 95 | 11 | 21 | 2 | 3 |
| Ware | 0 | 25 | 3 | 3 | 0 | 1 |
| Wareham | 0 | 37 | 10 | 12 | 1 | 2 |
| Warren | 0 | 13 | 1 | 1 | 0 | 0 |
| Warwick | 0 | 0 | 0 | 0 | 0 | 0 |
| Washington | 0 | 0 | 0 | 0 | 0 | 0 |
| Watertown | 2 | 33 | 8 | 11 | 1 | 1 |
| Wayland | 0 | 32 | 3 | 3 | 1 | 0 |
| Webster | 1 | 45 | 8 | 11 | 1 | 2 |
| Wellesley | 1 | 71 | 7 | 5 | 1 | 1 |
| Wellfleet | 0 | 0 | 1 | 1 | 0 | 0 |
| Wendell | 0 | 0 | 0 | 1 | 0 | 0 |
| Wenham | 0 | 6 | 1 | 1 | 0 | 0 |
| West Boylston | 0 | 16 | 2 | 4 | 0 | 0 |
| West Bridgewater | 0 | 14 | 4 | 3 | 0 | 0 |
| West Brookfield | 0 | 9 | 1 | 2 | 0 | 0 |
| West Newbury | 0 | 5 | 0 | 0 | 0 | 0 |
| West Springfield | 2 | 61 | 7 | 13 | 1 | 2 |
| West Stockbridge | 0 | 3 | 0 | 1 | 0 | 0 |
| West Tisbury | 0 | 4 | 1 | 1 | 0 | 0 |
| Westborough | 1 | 48 | 6 | 5 | 1 | 1 |
| Westfield | 2 | 80 | 12 | 16 | 1 | 4 |
| Westford | 0 | 71 | 4 | 5 | 0 | 1 |
| Westhampton | 0 | 3 | 0 | 0 | 0 | 0 |
| Westminster | 1 | 21 | 2 | 2 | 0 | 0 |
| Weston | 0 | 34 | 5 | 3 | 0 | 0 |
| Westport | 0 | 34 | 5 | 6 | 0 | 1 |
| Westwood | 1 | 53 | 8 | 6 | 1 | 1 |
| Weymouth | 3 | 104 | 16 | 25 | 1 | 4 |
| Whately | 0 | 0 | 0 | 0 | 0 | 0 |
| Whitman | 1 | 29 | 3 | 6 | 0 | 1 |
| Wilbraham | 0 | 25 | 3 | 3 | 0 | 0 |
| Williamsburg | 0 | 6 | 1 | 1 | 0 | 0 |
| Williamstown | 0 | 5 | 2 | 2 | 1 | 0 |
| Wilmington | 1 | 56 | 5 | 8 | 0 | 1 |
| Winchendon | 1 | 14 | 3 | 3 | 1 | 1 |
| Winchester | 1 | 73 | 6 | 6 | 2 | 0 |
| Windsor | 0 | 0 | 0 | 0 | 0 | 0 |
| Winthrop | 1 | 27 | 6 | 10 | 1 | 1 |
| Woburn | 2 | 67 | 11 | 19 | 2 | 4 |
| Worcester | 13 | 640 | 52 | 69 | 6 | 15 |
| Worthington | 0 | 0 | 1 | 1 | 0 | 0 |
| Wrentham | 0 | 21 | 3 | 3 | 0 | 1 |
| Yarmouth | 1 | 23 | 13 | 15 | 2 | 3 |

**Table 7. Estimated Performance IQ (PIQ) Points Lost due to PM_2.5_ Air Pollution by City and Town, Massachusetts, 2019**

| **Municipality** | **Child Population**  **(<10 years)** | **P1Q Point Loss Attributable to PM_2.5_ Air Pollution** | **Mean PIQ Point Loss**  **per Child** |
| --- | --- | --- | --- |
| Abington | 1450 | 3408 | 2.35 |
| Acton | 2586 | 7991 | 3.09 |
| Acushnet | 1083 | 3076 | 2.84 |
| Adams | 706 | 1850 | 2.62 |
| Agawam | 2751 | 6960 | 2.53 |
| Alford | 33 | 86 | 2.61 |
| Amesbury | 1572 | 3600 | 2.29 |
| Amherst | 1730 | 3962 | 2.29 |
| Andover | 4046 | 9265 | 2.29 |
| Aquinnah | 95 | 223 | 2.35 |
| Arlington | 6072 | 18762 | 3.09 |
| Ashburnham | 906 | 2039 | 2.25 |
| Ashby | 280 | 865 | 3.09 |
| Ashfield | 112 | 258 | 2.30 |
| Ashland | 2350 | 7262 | 3.09 |
| Athol | 1467 | 3301 | 2.25 |
| Attleboro | 5194 | 14751 | 2.84 |
| Auburn | 1579 | 3553 | 2.25 |
| Avon | 369 | 1026 | 2.78 |
| Ayer | 1081 | 3340 | 3.09 |
| Barnstable | 3654 | 8587 | 2.35 |
| Barre | 529 | 1190 | 2.25 |
| Becket | 155 | 349 | 2.25 |
| Bedford | 1708 | 5278 | 3.09 |
| Belchertown | 1658 | 3797 | 2.29 |
| Bellingham | 2079 | 5780 | 2.78 |
| Belmont | 3453 | 10670 | 3.09 |
| Berkley | 732 | 2079 | 2.84 |
| Berlin | 292 | 657 | 2.25 |
| Bernardston | 159 | 366 | 2.30 |
| Beverly | 3848 | 8812 | 2.29 |
| Billerica | 4194 | 12959 | 3.09 |
| Blackstone | 797 | 1793 | 2.25 |
| Blandford | 59 | 149 | 2.53 |
| Bolton | 707 | 1591 | 2.25 |
| Boston | 64052 | 217136 | 3.39 |
| Bourne | 1436 | 3375 | 2.35 |
| Boxborough | 575 | 1777 | 3.09 |
| Boxford | 1051 | 2407 | 2.29 |
| Boylston | 440 | 990 | 2.25 |
| Braintree | 4571 | 12707 | 2.78 |
| Brewster | 626 | 1471 | 2.35 |
| Bridgewater | 2678 | 6293 | 2.35 |
| Brimfield | 437 | 1106 | 2.53 |
| Brockton | 13166 | 30940 | 2.35 |
| Brookfield | 324 | 729 | 2.25 |
| Brookline | 6060 | 16847 | 2.78 |
| Buckland | 184 | 423 | 2.30 |
| Burlington | 3018 | 9326 | 3.09 |
| Cambridge | 8841 | 27319 | 3.09 |
| Canton | 2875 | 7993 | 2.78 |
| Carlisle | 540 | 2106 | 3.90 |
| Carver | 1074 | 2524 | 2.35 |
| Charlemont | 85 | 196 | 2.31 |
| Charlton | 1756 | 3951 | 2.25 |
| Chatham | 342 | 804 | 2.35 |
| Chelmsford | 3913 | 12091 | 3.09 |
| Chelsea | 6187 | 20974 | 3.39 |
| Cheshire | 179 | 469 | 2.62 |
| Chester | 164 | 415 | 2.53 |
| Chesterfield | 103 | 236 | 2.29 |
| Chicopee | 5989 | 15152 | 2.53 |
| Chilmark | 51 | 120 | 2.35 |
| Clarksburg | 195 | 511 | 2.62 |
| Clinton | 1356 | 3051 | 2.25 |
| Cohasset | 1354 | 3764 | 2.78 |
| Colrain | 179 | 412 | 2.30 |
| Concord | 2226 | 6878 | 3.09 |
| Conway | 140 | 322 | 2.30 |
| Cummington | 76 | 174 | 2.29 |
| Dalton | 525 | 1376 | 2.62 |
| Danvers | 3004 | 6879 | 2.29 |
| Dartmouth | 2352 | 6680 | 2.84 |
| Dedham | 2631 | 7314 | 2.78 |
| Deerfield | 480 | 1104 | 2.30 |
| Dennis | 1150 | 2703 | 2.35 |
| Dighton | 605 | 1718 | 2.84 |
| Douglas | 762 | 1715 | 2.25 |
| Dover | 740 | 2057 | 2.78 |
| Dracut | 3660 | 11309 | 3.09 |
| Dudley | 1060 | 2385 | 2.25 |
| Dunstable | 249 | 769 | 3.09 |
| Duxbury | 1825 | 4289 | 2.35 |
| East Bridgewater | 2087 | 4904 | 2.35 |
| East Brookfield | 357 | 803 | 2.25 |
| East Longmeadow | 1748 | 4422 | 2.53 |
| Eastham | 231 | 543 | 2.35 |
| Easthampton | 1267 | 2901 | 2.29 |
| Easton | 2445 | 6944 | 2.84 |
| Edgartown | 342 | 804 | 2.35 |
| Egremont | 68 | 178 | 2.62 |
| Erving | 173 | 398 | 2.30 |
| Essex | 450 | 1031 | 2.29 |
| Everett | 6626 | 20474 | 3.09 |
| Fairhaven | 1711 | 4859 | 2.84 |
| Fall River | 11321 | 32152 | 2.84 |
| Falmouth | 2864 | 6730 | 2.35 |
| Fitchburg | 4869 | 10955 | 2.25 |
| Florida | 55 | 144 | 2.62 |
| Foxborough | 2081 | 5785 | 2.78 |
| Framingham | 7684 | 23744 | 3.09 |
| Franklin | 3724 | 10353 | 2.78 |
| Freetown | 979 | 2780 | 2.84 |
| Gardner | 2280 | 5130 | 2.25 |
| Georgetown | 847 | 1940 | 2.29 |
| Gill | 96 | 221 | 2.30 |
| Gloucester | 2716 | 6220 | 2.29 |
| Goshen | 105 | 240 | 2.29 |
| Gosnold | 0 | 0 | 0 |
| Grafton | 2077 | 4673 | 2.25 |
| Granby | 583 | 1335 | 2.29 |
| Granville | 195 | 493 | 2.53 |
| Great Barrington | 442 | 1158 | 2.62 |
| Greenfield | 1658 | 3813 | 2.30 |
| Groton | 1315 | 4063 | 3.09 |
| Groveland | 699 | 1601 | 2.29 |
| Hadley | 333 | 763 | 2.29 |
| Halifax | 952 | 2237 | 2.35 |
| Hamilton | 1076 | 2464 | 2.29 |
| Hampden | 427 | 1080 | 2.53 |
| Hancock | 47 | 123 | 2.62 |
| Hanover | 2024 | 4756 | 2.35 |
| Hanson | 803 | 1887 | 2.35 |
| Hardwick | 374 | 842 | 2.25 |
| Harvard | 652 | 1467 | 2.25 |
| Harwich | 678 | 1593 | 2.35 |
| Hatfield | 114 | 261 | 2.29 |
| Haverhill | 8285 | 18973 | 2.29 |
| Hawley | 36 | 83 | 2.31 |
| Heath | 95 | 219 | 2.31 |
| Hingham | 3158 | 7421 | 2.35 |
| Hinsdale | 141 | 369 | 2.62 |
| Holbrook | 876 | 2435 | 2.78 |
| Holden | 2227 | 5011 | 2.25 |
| Holland | 233 | 589 | 2.53 |
| Holliston | 1756 | 5426 | 3.09 |
| Holyoke | 5568 | 14087 | 2.53 |
| Hopedale | 516 | 1161 | 2.25 |
| Hopkinton | 2225 | 6875 | 3.09 |
| Hubbardston | 421 | 947 | 2.25 |
| Hudson | 1739 | 5374 | 3.09 |
| Hull | 630 | 1481 | 2.35 |
| Huntington | 162 | 371 | 2.29 |
| Ipswich | 1311 | 3002 | 2.29 |
| Kingston | 1499 | 3523 | 2.35 |
| Lakeville | 1105 | 2597 | 2.35 |
| Lancaster | 635 | 1429 | 2.25 |
| Lanesborough | 479 | 1255 | 2.62 |
| Lawrence | 11830 | 27091 | 2.29 |
| Lee | 489 | 1281 | 2.62 |
| Leicester | 1083 | 2437 | 2.25 |
| Lenox | 349 | 914 | 2.62 |
| Leominster | 5273 | 11864 | 2.25 |
| Leverett | 182 | 419 | 2.30 |
| Lexington | 3890 | 12020 | 3.09 |
| Leyden | 48 | 110 | 2.29 |
| Lincoln | 939 | 2902 | 3.09 |
| Littleton | 1230 | 3801 | 3.09 |
| Longmeadow | 1780 | 4503 | 2.53 |
| Lowell | 14212 | 43915 | 3.09 |
| Ludlow | 1921 | 4860 | 2.53 |
| Lunenburg | 986 | 2219 | 2.25 |
| Lynn | 13399 | 30684 | 2.29 |
| Lynnfield | 1732 | 3966 | 2.29 |
| Malden | 6725 | 20780 | 3.09 |
| Manchester-by-the-Sea | 424 | 971 | 2.29 |
| Mansfield | 2667 | 7574 | 2.84 |
| Marblehead | 2274 | 5207 | 2.29 |
| Marion | 552 | 1297 | 2.35 |
| Marlborough | 4260 | 13163 | 3.09 |
| Marshfield | 2935 | 6897 | 2.35 |
| Mashpee | 1108 | 2604 | 2.35 |
| Mattapoisett | 391 | 919 | 2.35 |
| Maynard | 1307 | 4039 | 3.09 |
| Medfield | 1970 | 5477 | 2.78 |
| Medford | 5259 | 20510 | 3.90 |
| Medway | 1493 | 4151 | 2.78 |
| Melrose | 3542 | 10945 | 3.09 |
| Mendon | 450 | 1013 | 2.25 |
| Merrimac | 611 | 1399 | 2.29 |
| Methuen | 5340 | 12229 | 2.29 |
| Middleborough | 2571 | 6042 | 2.35 |
| Middlefield | 18 | 41 | 2.28 |
| Middleton | 921 | 2109 | 2.29 |
| Milford | 4007 | 9016 | 2.25 |
| Millbury | 1528 | 3438 | 2.25 |
| Millis | 753 | 2093 | 2.78 |
| Millville | 385 | 866 | 2.25 |
| Milton | 3940 | 10953 | 2.78 |
| Monroe | 11 | 25 | 2.27 |
| Monson | 794 | 2009 | 2.53 |
| Montague | 767 | 1764 | 2.30 |
| Monterey | 35 | 92 | 2.63 |
| Montgomery | 56 | 142 | 2.54 |
| Mount Washington | 0 | 0 | 0 |
| Nahant | 138 | 316 | 2.29 |
| Nantucket | 1245 | 2926 | 2.35 |
| Natick | 4910 | 15172 | 3.09 |
| Needham | 4085 | 11356 | 2.78 |
| New Ashford | 15 | 39 | 2.60 |
| New Bedford | 12144 | 31817 | 2.62 |
| New Braintree | 144 | 324 | 2.25 |
| New Marlborough | 63 | 165 | 2.62 |
| New Salem | 98 | 225 | 2.30 |
| Newbury | 483 | 1106 | 2.29 |
| Newburyport | 1698 | 3888 | 2.29 |
| Newton | 9592 | 29639 | 3.09 |
| Norfolk | 1340 | 3725 | 2.78 |
| North Adams | 1124 | 2945 | 2.62 |
| North Andover | 3939 | 9020 | 2.29 |
| North Attleborough | 3304 | 9383 | 2.84 |
| North Brookfield | 558 | 1256 | 2.25 |
| North Reading | 1899 | 5868 | 3.09 |
| Northampton | 2364 | 5414 | 2.29 |
| Northborough | 1737 | 3908 | 2.25 |
| Northbridge | 2319 | 5218 | 2.25 |
| Northfield | 274 | 630 | 2.30 |
| Norton | 1854 | 4264 | 2.30 |
| Norwell | 1512 | 3553 | 2.35 |
| Norwood | 3268 | 9085 | 2.78 |
| Oak Bluffs | 688 | 1617 | 2.35 |
| Oakham | 119 | 268 | 2.25 |
| Orange | 782 | 1799 | 2.30 |
| Orleans | 328 | 771 | 2.35 |
| Otis | 101 | 265 | 2.62 |
| Oxford | 1775 | 3994 | 2.25 |
| Palmer | 1036 | 2621 | 2.53 |
| Paxton | 540 | 1215 | 2.25 |
| Peabody | 5098 | 11674 | 2.29 |
| Pelham | 146 | 334 | 2.29 |
| Pembroke | 1922 | 4517 | 2.35 |
| Pepperell | 1515 | 4681 | 3.09 |
| Peru | 68 | 178 | 2.62 |
| Petersham | 86 | 194 | 2.26 |
| Phillipston | 177 | 398 | 2.25 |
| Pittsfield | 4501 | 11793 | 2.62 |
| Plainfield | 52 | 119 | 2.29 |
| Plainville | 772 | 2146 | 2.78 |
| Plymouth | 5420 | 12737 | 2.35 |
| Plympton | 324 | 761 | 2.35 |
| Princeton | 288 | 648 | 2.25 |
| Provincetown | 75 | 176 | 2.35 |
| Quincy | 8531 | 23716 | 2.78 |
| Randolph | 3419 | 9505 | 2.78 |
| Raynham | 1714 | 2028 | 1.18 |
| Reading | 3212 | 9925 | 3.09 |
| Rehoboth | 1109 | 3150 | 2.84 |
| Revere | 6194 | 20998 | 3.39 |
| Richmond | 115 | 301 | 2.62 |
| Rochester | 554 | 1302 | 2.35 |
| Rockland | 1958 | 4601 | 2.35 |
| Rockport | 590 | 1351 | 2.29 |
| Rowe | 25 | 58 | 2.32 |
| Rowley | 660 | 1511 | 2.29 |
| Royalston | 43 | 97 | 2.26 |
| Russell | 193 | 488 | 2.53 |
| Rutland | 1008 | 2268 | 2.25 |
| Salem | 4115 | 9423 | 2.29 |
| Salisbury | 814 | 1864 | 2.29 |
| Sandisfield | 105 | 275 | 2.62 |
| Sandwich | 1780 | 4183 | 2.35 |
| Saugus | 2524 | 5780 | 2.29 |
| Savoy | 48 | 126 | 2.63 |
| Scituate | 1911 | 4491 | 2.35 |
| Seekonk | 1217 | 3456 | 2.84 |
| Sharon | 2246 | 6244 | 2.78 |
| Sheffield | 230 | 603 | 2.62 |
| Shelburne | 112 | 258 | 2.30 |
| Sherborn | 557 | 1721 | 3.09 |
| Shirley | 487 | 1505 | 3.09 |
| Shrewsbury | 4502 | 10130 | 2.25 |
| Shutesbury | 149 | 343 | 2.30 |
| Somerset | 1554 | 4413 | 2.84 |
| Somerville | 5860 | 18107 | 3.09 |
| South Hadley | 1419 | 3250 | 2.29 |
| Southampton | 539 | 1234 | 2.29 |
| Southborough | 1319 | 2968 | 2.25 |
| Southbridge | 1972 | 4437 | 2.25 |
| Southwick | 1082 | 2737 | 2.53 |
| Spencer | 896 | 2016 | 2.25 |
| Springfield | 20926 | 52943 | 2.53 |
| Sterling | 813 | 1829 | 2.25 |
| Stockbridge | 139 | 364 | 2.62 |
| Stoneham | 2239 | 8732 | 3.90 |
| Stoughton | 2815 | 7826 | 2.78 |
| Stow | 954 | 2948 | 3.09 |
| Sturbridge | 1248 | 2808 | 2.25 |
| Sudbury | 2522 | 7793 | 3.09 |
| Sunderland | 266 | 612 | 2.30 |
| Sutton | 808 | 1818 | 2.25 |
| Swampscott | 1944 | 4452 | 2.29 |
| Swansea | 1540 | 4374 | 2.84 |
| Taunton | 6633 | 18838 | 2.84 |
| Templeton | 720 | 1620 | 2.25 |
| Tewksbury | 2687 | 10479 | 3.90 |
| Tisbury | 228 | 536 | 2.35 |
| Tolland | 37 | 94 | 2.54 |
| Topsfield | 812 | 1859 | 2.29 |
| Townsend | 1271 | 3927 | 3.09 |
| Truro | 61 | 143 | 2.34 |
| Tyngsborough | 1200 | 4680 | 3.90 |
| Tyringham | 29 | 76 | 2.62 |
| Upton | 894 | 2012 | 2.25 |
| Uxbridge | 1473 | 3314 | 2.25 |
| Wakefield | 2571 | 10027 | 3.90 |
| Wales | 259 | 655 | 2.53 |
| Walpole | 3067 | 8526 | 2.78 |
| Waltham | 5722 | 17681 | 3.09 |
| Ware | 980 | 2244 | 2.29 |
| Wareham | 2361 | 5548 | 2.35 |
| Warren | 515 | 1159 | 2.25 |
| Warwick | 63 | 145 | 2.30 |
| Washington | 54 | 141 | 2.61 |
| Watertown | 3461 | 10694 | 3.09 |
| Wayland | 1982 | 6124 | 3.09 |
| Webster | 2039 | 4588 | 2.25 |
| Wellesley | 3504 | 9741 | 2.78 |
| Wellfleet | 144 | 338 | 2.35 |
| Wendell | 84 | 193 | 2.30 |
| Wenham | 443 | 1014 | 2.29 |
| West Boylston | 574 | 1292 | 2.25 |
| West Bridgewater | 807 | 1896 | 2.35 |
| West Brookfield | 327 | 736 | 2.25 |
| West Newbury | 499 | 1143 | 2.29 |
| West Springfield | 3282 | 8303 | 2.53 |
| West Stockbridge | 87 | 228 | 2.62 |
| West Tisbury | 82 | 193 | 2.35 |
| Westborough | 2555 | 5749 | 2.25 |
| Westfield | 3895 | 9854 | 2.53 |
| Westford | 2698 | 8876 | 3.29 |
| Westhampton | 184 | 421 | 2.29 |
| Westminster | 849 | 1910 | 2.25 |
| Weston | 1666 | 5148 | 3.09 |
| Westport | 1248 | 5112 | 4.10 |
| Westwood | 2081 | 5785 | 2.78 |
| Weymouth | 5845 | 16249 | 2.78 |
| Whately | 125 | 288 | 2.30 |
| Whitman | 1762 | 4141 | 2.35 |
| Wilbraham | 1258 | 3183 | 2.53 |
| Williamsburg | 167 | 382 | 2.29 |
| Williamstown | 568 | 152 | 0.27 |
| Wilmington | 2653 | 8198 | 3.09 |
| Winchendon | 1447 | 3256 | 2.25 |
| Winchester | 3256 | 10061 | 3.09 |
| Windsor | 58 | 152 | 2.62 |
| Winthrop | 1668 | 5655 | 3.39 |
| Woburn | 4173 | 12895 | 3.09 |
| Worcester | 19276 | 43371 | 2.25 |
| Worthington | 93 | 213 | 2.29 |
| Wrentham | 1498 | 4164 | 2.78 |
| Yarmouth | 1843 | 4331 | 2.35 |
| **TOTAL** | **732,596** | **1,999,639** | **2.73** |
